# Acidification-Induced Structure Evolution of Lipid Nanoparticles Correlates with Their *In Vitro* Gene Transfections

**DOI:** 10.1021/acsnano.2c06213

**Published:** 2023-01-06

**Authors:** Zongyi Li, Jessica Carter, Luis Santos, Carl Webster, Christopher F. van der Walle, Peixun Li, Sarah E. Rogers, Jian Ren Lu

**Affiliations:** † Biological Physics Laboratory, School of Physics and Astronomy, University of Manchester, Oxford Road, Schuster Building, ManchesterM13 9PL, U.K.; ‡ Dosage Form Design Development, Biopharmaceuticals Development, AstraZeneca, Gaithersburg, Maryland20878, United States; § Discovery Sciences, R&D, AstraZeneca, CambridgeCB21 6GH, U.K.; ∥ The Cell and Gene Therapy Catapult, The Centre for Regenerative Medicine, 5 Little France Drive, EdinburghEH16 4UU, U.K.; ⊥ ISIS Neutron Facility, STFC, Chilton, DidcotOX11 0QZ, U.K.

**Keywords:** lipid nanoparticles, nonviral gene delivery, small-angle neutron scattering, cellular expression, acidification-induced structure evolution (AISE)

## Abstract

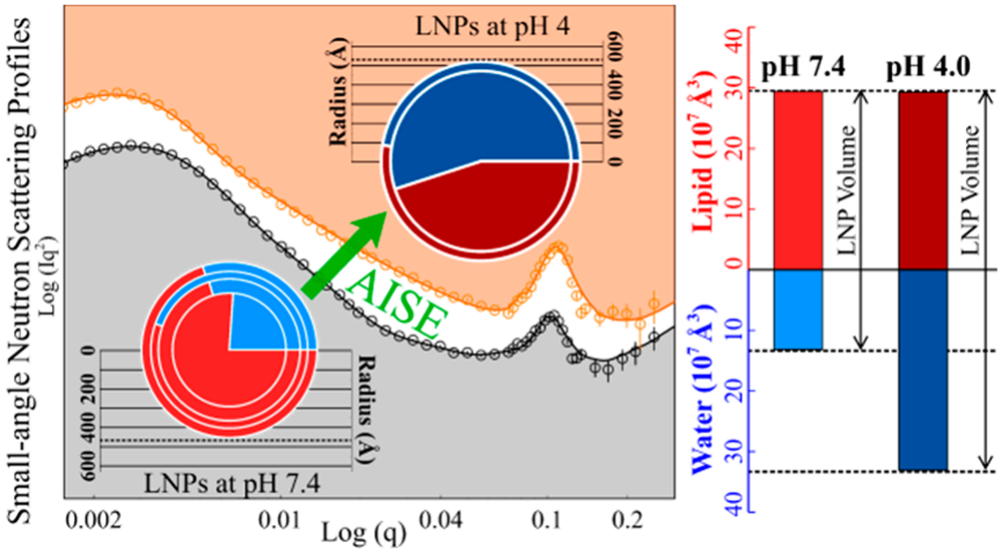

The rational design of lipid nanoparticles (LNPs) for enhanced gene delivery remains challenging because of incomplete knowledge of their formulation–structure relationship that impacts their intracellular behavior and consequent function. Small-angle neutron scattering has been used in this work to investigate the structure of LNPs encapsulating plasmid DNA upon their acidification (from pH 7.4 to 4.0), as would be encountered during endocytosis. The results revealed the acidification-induced structure evolution (AISE) of the LNPs on different dimension scales, involving protonation of the ionizable lipid, volume expansion and redistribution of aqueous and lipid components. A similarity analysis using an LNP’s structural feature space showed a strong positive correlation between function (measured by intracellular luciferase expression) and the extent of AISE, which was further enhanced by the fraction of unsaturated helper lipid. Our findings reveal molecular and nanoscale changes occurring during AISE that underpin the LNPs’ formulation–nanostructure–function relationship, aiding the rational design of application-directed gene delivery vehicles.

## Introduction

Gene delivery offers solutions for the treatment of rare diseases and, more recently, viral pandemics. An exemplary vector will protect the nucleic acid payload from degradation and overcome a series of extracellular and intracellular barriers to enable efficient gene translation/transcription at the desired cell/tissue target.^1,2^ Lipid nanoparticle (LNP) vectors formulated with an ionizable lipid, a PEGylated lipid, cholesterol, and a helper lipid have demonstrated clinical efficacy and safety in disease prevention and treatment,^3−5^ most recently as vectors of mRNA vaccines.^6,7^ Their rapid adoption is in no small part due to decades of earlier research^8−10^ and the prior approval of Onpattro.^11^

One of the major challenges in developing LNP-based technologies is finding optimized lipid formulations for enhancing protein production, which are typically target-specific, differing between various cells/tissues. Extensive efforts have been made on tuning the components’ chemical structures and mixing proportions to enhance the LNP’s delivery efficiency in different applications.^4,12−15^ Changes in lipid type and composition alter their interactions and result in different nanostructures such as inverted micelles and inverted-hexagonal, multiple-lamellar and amorphous phases for different formulations.^16^ Moreover, the LNP nanostructure is related to its capacity for cellular uptake and release of the payload.^17,18^ However, LNP development studies must rely on high-throughput screening work *in vitro* and *in vivo* due to the nature of multiple components and the complexity of LNP manufacturing. Thus, the rational design of LNP carriers, in the context of linking the structural features of key components to their functional roles and proposing target-specific adjustment, is a step forward for future LNP-based gene delivery applications.

The path to achieving the rational design of LNP carriers requires a better understanding of the LNP structures, which is the core of the LNP’s formulation–structure–function relationship. The formulation mechanism of lipid nanocarriers and the structural phase transitions occurring upon encapsulation of nucleic acids have been previously investigated by synchrotron small-angle X-ray scattering (SAXS)^19,20^ and the component distributions across the LNP by SANS.^21,22^ Various LNP structure models have been proposed by different techniques.^17,23^ However, to date, most studies have been done at physiological pH, thereby omitting essential information on the effect of acidification, e.g., during the maturation of endosomes;^24^ the endosomal escape process is widely regarded as the bottleneck for LNP applications to achieve high transfection efficiencies.^25−27^ Furthermore, the p*K*_a_ values of the ionizable lipids have been shown to play a crucial role in LNP function.^5^ Hassett et al.^28^ suggested that the optimal p*K*_a_ value of the ionizable lipids alters in different administration routes, while Rybak and Murphy^29^ have revealed that different cells have different endosomal acidifications. Thus, investigating how LNPs behave under acidic pH conditions becomes especially pertinent.

In this work, we investigated the structures of different plasmid DNA (pDNA)-LNPs in both physiological (pH 7.4) and mildly acidic (pH 4) environments. The morphological features, including size, shape, hydration, and lipid distribution, and the internal structure of the LNPs were investigated using a combination of techniques of dynamic light scattering (DLS), transmission electron cryomicroscopy (cryo-TEM), and small-angle neutron scattering (SANS) with isotopic contrast variation. Notably, a modified SANS model is herein described which captures structural features and their relationship on different length scales, from a few nanometers to a few hundred nanometers, generating data complementary to cryo-TEM images. The data analysis approach required to support the model uses the LNP’s structural feature space (LSF) to compare LNPs and numerically evaluate their similarity. In doing so, we overcame current challenges in comparing different LNP formulations based on the multiply measured properties required.

The most striking observation in this work is the phenomena of the acidification-induced structure evolution (AISE) of the LNPs, during which all the LNPs investigated underwent a volume expansion process, accompanied by a redistribution of LNP’s components and nanostructures, revealing structural transformation on different length scales. We also examined how the LNP’s morphology and the AISE process were influenced by the LNP payloads, which helped reveal the mechanism of this strong structural responsiveness of LNPs to pH. Furthermore, we observed a strong correlation between the extent of AISE and *in vitro* transfection efficacy of LNPs. Moreover, we found that the helper lipid could play a pivotal role in affecting the extent of AISE, even though the helper lipid is only 10% (molar ratio) of the total LNP components. By altering the helper lipids, we revealed that helper lipids with an unsaturated acyl chain could increase the extent of AISE and result in a higher *in vitro* transfection efficacy.

## Results and Discussion

### Production and Key Functional and Structural Features of SOPC_pLuc LNP

**Figure 1:**
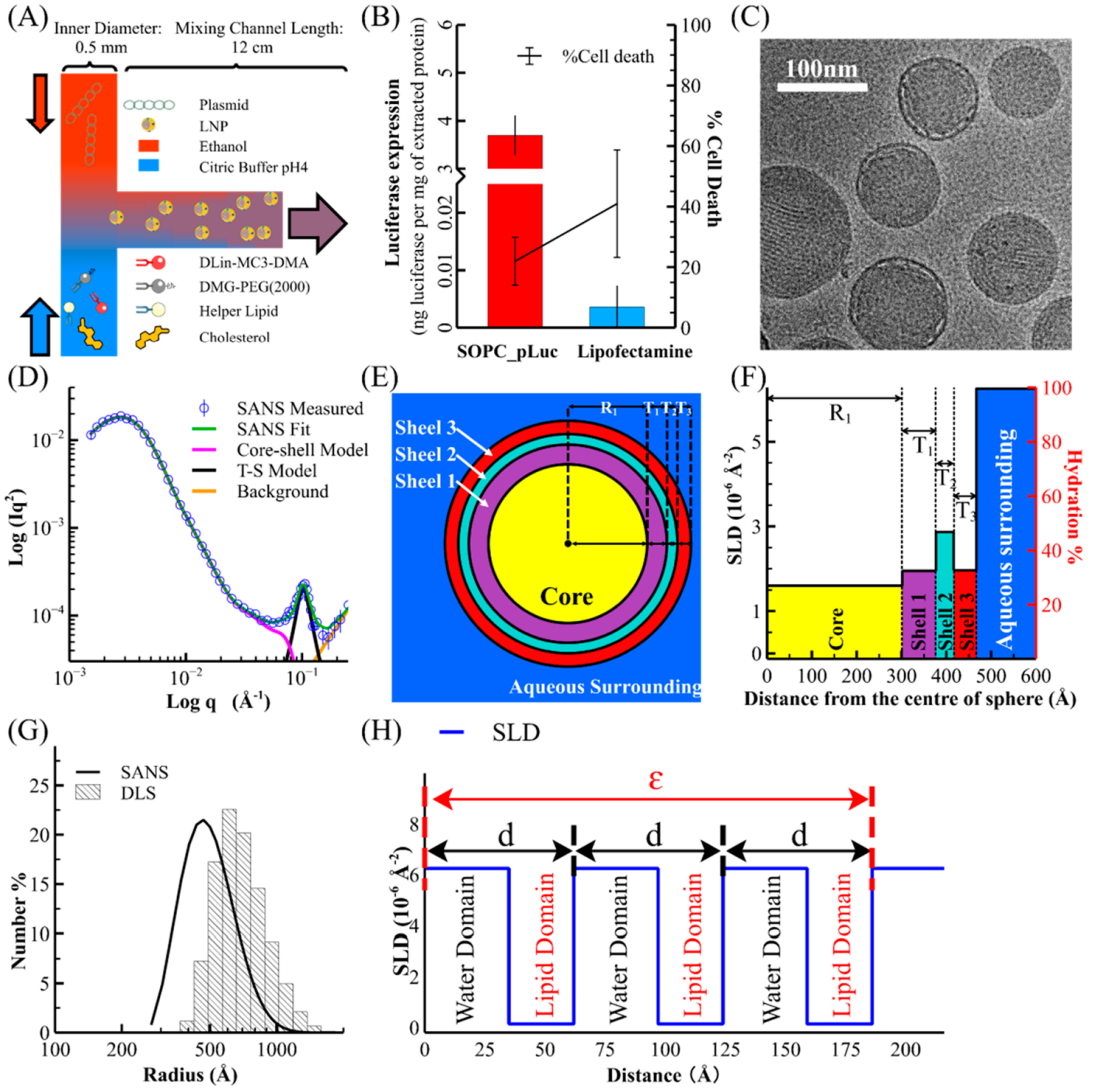
LNP production, key functional and structural features. (A) Schematic diagram illustrating the fabrication of plasmid-containing LNPs via the FNC method using a confined impinging jet device based on a T-junction mixer. (B) Transfection efficiency and toxicity (percentage cell death) of the SOPC_pLuc LNPs applied to Hela cells at the captured plasmid concentration of 100 ng/mL (results for other concentrations are given in SI 4). Lipofectamine 2000 transfection reagent was used as a positive control. (C) A cryo-TEM image of the SOPC_pLuc LNPs. More images are given in SI 3. (D) The measured SANS profile of SOPC_pLuc LNP at pH 7.4 (pD 7.7 in PBS D_2_O buffer) plotted in the form of the Kratky plot of Log(*Iq*2) against Log(*q*), where *I* denotes the scattering intensity. (E) Schematic representation of the core–shells model. The core, Shell 1 (inner shell), Shell 2 (mid shell), Shell 3 (outer shell), and the aqueous surrounding are labeled with yellow, purple, teal, red, and blue, respectively. (F) Radial SLD and hydration (water volume fraction) profile of the LNP associated with the core–shells model in (E). (G) The LNPs’ size distribution is measured by SANS (black line) and DLS (histogram). (H) Schematic diagram showing a 1D SLD periodic fluctuation raised from two alternating domains (lipid domain and water domain).

The LNPs used in this study were prepared using the flash nanocomplexation (FNC) method, which was reported to produce highly uniform PEI/DNA complex nanoparticles,^30^ lipoplexes,^31^ and LNPs^32^ on a large scale with high reproducibility.^33^Figure 1(A) shows the schematic of the FNC method. The mixing device was based on a T-junction mixer (Figure S2), allowing the rapid mixing of two impinging fluid streams, water, and ethanol. The water stream contained the nucleic acid in citrate buffer pH 4, while all lipids were predissolved in ethanol as the second stream. We chose a “benchmark” LNP formulation for this study, which was widely used in literature.^11,21,22,34−37^ The lipid compositions of DLin-MC3-DMA (MC3):cholesterol:helper lipid:ethoxylate lipid DMG-PEG(2000) were fixed at a molar ratio of 50:38.5:10:1.5, respectively, and the N/P ratio during the two-stream mixing process was fixed at 6:1. l-α-1-Stearoyl-2-oleoylphosphatidylcholine (SOPC) was used as the helper lipid to generate our first LNP sample encapsulating the luciferase-encoding plasmid, pLuc (5.5k bps), referred to as SOPC_pLuc LNPs. A screening process helped optimize the production parameters, including sample concentrations and streamflow rates, to obtain the lowest polydispersity index of the size distribution (PDI < 0.1) and the highest nucleic acid encapsulation rate (>90%). Detailed information about the preparation and optimization of LNPs are given in sections SI 1 and 2 of the Supporting Information.

We investigated the *in vitro* protein expression efficiency and toxicity of the LNPs using Hela cells, maintained in Dulbecco’s modified Eagle’s medium (DMEM) supplemented with 10% fetal bovine serum (FBS). Figure 1(B) shows that the SOPC_pLuc LNPs have almost a 100-fold improvement in transfection efficiency for half the percentage of cell death against Lipofectamine 2000 used as the control.

The cryo-TEM images (Figure 1(C) and SI 3) show that the SOPC_pLuc LNPs obtained are largely spherical, fairly uniform, and have an electron-dense core enfolded in a continuum outer-membrane shell, similar to the morphologies reported previously.^22,35,38^

Figure 1(D) shows the SANS profile of the SOPC_pLuc LNPs measured in phosphate buffered saline (PBS) D_2_O buffer (pD 7.7 equiv to pH 7.4 in H_2_O). The green line is the best fit using a combined model, which is the sum of the core–shells model (Magenta line), the Teubner–Strey (T–S) model (Black line), and a constant background (orange line). The core–shells model describes the “bell shape” peak over the low q range, which indicates an overall spherical shape on a length scale matching the diameters of the LNPs, consistent with the TEM observations. The T–S model allows the quantitative description of the LNP’s nanostructure on the length scale of a few nanometers, which corresponds to the peak feature at the *q* position of about 0.1 Å^–1^. The T–S model is a shape-independent model derived from Landau’s free energy theory to describe the scattering intensity from a binary component system with a quasi-periodic structure.^39^ It was widely used to describe microemulsion systems adopting structures with different levels of disorder, such as bicontinuous network and lamellar structure.^40^

A schematic of the core–shells model is shown in Figure 1(E), illustrating a spherical core and its concentric shells that subdivide the LNP into different regions by distinct scattering length densities (SLDs). The SLD value can be converted to the hydration (or water volume fraction) using eq 1
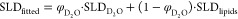
1where φ_D_2_O_ is the water volume fraction. Here, the LNPs are simplified as a binary-component system containing the water and lipid components. The lipid component includes all the lipids and cholesterol. This simplification is based on the fact that the protonated lipids and cholesterol have so close SLDs that their differences can be neglected. More details about the core–shells model and the calculation of the water volume fraction are given in SI 5. Since the core–shells model is geometrically symmetric, the model can be presented as a 1D radial SLD (primar*y*-axis) or hydration (secondar*y*-axis) profile, as shown in Figure 1(F). The outer shell (Shell 3) has a thickness of around 50 Å and contains 30% water and 70% lipids, corresponding to the continuum lipid bilayer as the outer membrane surrounding the LNP. The inner shell (Shell 1) has a close thickness and water volume fraction to the outer shell. The mid shell (Shell 2) has the highest water volume fraction, sandwiched between the outer shell and inner shell, commensurate with the water-rich region observed in the cryo-TEM image shown in Figure 1(C). This water-rich shell has been reported and is regarded as evidence of the phase segregating from the LNP outer shell and core in a previous study by cryo-TEM.^35^ Previous studies have reported a similar electron-dense core observed in the cryo-TEM images of LNP-siRNA systems,^38,41^ and the core was assumed to be an amorphous oil droplet phase formed from the neutral ionizable lipid so that the hydrophilic plasmid can only be trapped in the water-rich region (mid shell). Our SANS result suggested that the water volume fraction of the core region is about 24%. The presence of water indicates that the plasmid could also be in the core region.

The histogram in Figure 1(G) shows the LNP’s radius distribution determined by DLS, and the line distribution is the equivalent measured from SANS (further information is given in SI 6). The two radius distributions have a log-normal distribution shape with similar polydispersity but different center positions. The central value of the LNP size distribution measured by DLS is larger than that by SANS because the definitions of particle edge between these two techniques are different. SANS measures the particle’s “true” radius, in which the particle’s outer circumference is defined as the boundary between the material and the water surrounding it. DLS determines the hydrodynamic radius by measuring the particle’s diffusion coefficient, which can be significantly affected if there are PEG segments anchored on the LNP surface. Previous experimental^42^ and computer modeling^43^ studies have shown that the PEG-lipid is mainly located on the LNP surface, and the PEGylation can improve the LNP’s colloidal stability and circulation lifetime.^44^ Thus, DLS is more sensitive to the PEG region than SANS if the PEG region is sparse with a meager PEG volume fraction.

Although a single broad peak feature in reciprocal space is insufficient for ultimately defining a 3D structure in real space, the SANS peak feature at the *q* position about 0.1 Å^–1^ reveals a nanostructure with quasi-periodic SLD fluctuation as shown in the schematic diagram in Figure 1(H). This SLD fluctuation raised from the alternating lipid and water domains with distinct SLD values (SLD of D_2_O is 6.35 × 10^–6^ Å^–2^ and SLD of lipids is around 0.1 × 10^–6^ Å^–2^). The periodicity length (*d*) is this system’s average spatial period, which also equals the sum of averaged dimensions of these two domains. The structure is so-called quasi-periodic, which means this orientational periodicity is only preserved in a short distance on average. This averaged distance is called correlation length (ε), which equals three times *d* in the example shown in Figure 1(H). However, for the SOPC_pLuc LNPs at pH 7.4 (pD 7.7), Table 1 shows that the correlation length is 67.6 Å, close to the periodicity length (62 Å). The similarity of these two numbers implies that the nanostructure is not a highly ordered structure. The Cryo-TEM images corroborate this interpretation, showing no highly ordered structures (e.g., stacked lamella, or “onion-like” layers, or bicontinuous cubic phases as reported for LNP-siRNA systems elsewhere,^45−47^ or L_α_^C^, H_I_^C^, and H_II_^C^ phases reported in a series of cationic liposomes (CLs)–DNA systems.^48−50^)

**Table 1:** Changes in Structural Parameters of the SOPC LNPs from SANS and DLS Measurements^a^

	SANS					DLS		
pH	Radius (±6 Å)	Schulz Polydispersity (±0.02)	d (±0.5 Å)	ε (±0.5 Å)	γ (±2%)	Hydrodynamic Radius (±3 Å)	PDI (±0.01)	ZP (±3 mV)
7.4	467	0.53	62.0	67.7	–0.958	625	0.07	–26.6
4.0	530	0.41	59.1	68.9	–0.963	750	0.05	4.3

a The table covers the hydrodynamic radius, size polydispersity (PDI), and zeta potential (ZP) measured by DLS, LNP radius, periodicity thickness (*d*), correlation length (ε), and amphiphile strength (γ) from SANS data analysis.

In addition, a parameter called the amphiphile strength (γ) can be deduced from *d* and ε (more information is given in section SI 5), and its values can empirically imply the structural characteristics. For example, a lamellar structure has γ < −1, and −1 < γ < 0 corresponds to nonlamellar structures.^40^ For the SOPC_pLuc LNPs at pH 7.4 (pD 7.7), the amphiphile strength γ is −0.96, indicating a nanostructure close to the boundary between lamellar and nonlamellar phases.

### Acidification-Induced Structure Evolution (AISE) of SOPC_pLuc LNPs

**Figure 2:**
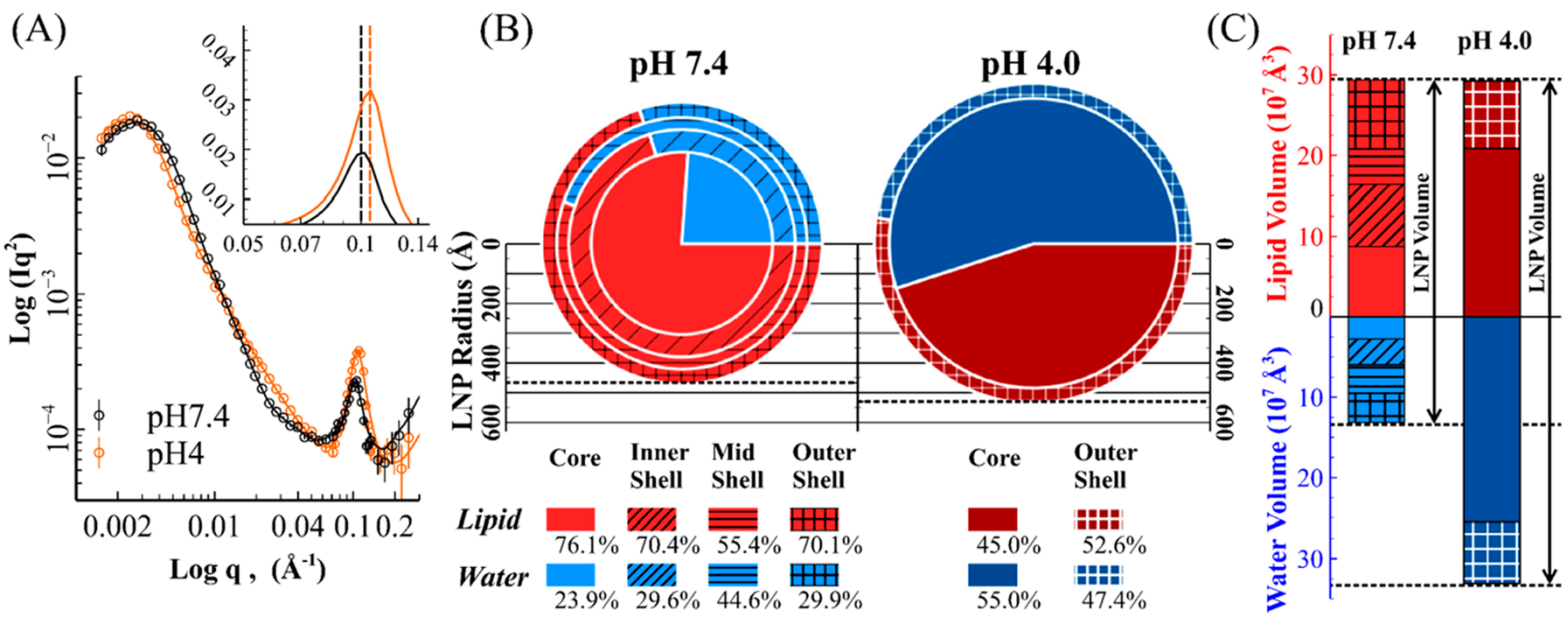
SANS analysis reveals the AISE of LNPs. (A) SANS profiles of SOPC_pLuc LNPs in D_2_O at physiological (pD 7.7, black symbols, equivalent to pH 7.4 in H_2_O) and acidic (pD 4.3, orange symbols, equivalent to pH 4.0 in H_2_O) conditions. The solid lines represent the best SANS fits from the combined models. The subgraph shows the T–S model contributions to the SANS fitting model. (B) Pie charts show the proportional changes of lipid mixture (red) and water (blue) in each region of the SOPC_pLuc LNPs at the two pHs as indicated in the legend, with the proportion values and the radii being obtained from best fits of the SANS data. (C) Stacked bar charts display the volume changes of lipid mixture (red) and water (blue), with core and shells being marked in the same patterns as in (B).

Figure 2(A) shows the SANS profiles of the SOPC_pLuc LNPs measured at pH 7.4 (PBS buffer 20 mM, 137 mM NaCl) and pH4 (citric buffer 20 mM, NaCl 137 mM). The sample preparation at different pHs is given in SI 2. The difference in the SANS profiles induced by acidification is visible across the whole *q* range, indicating that the structural evolution is related to the LNP features on both the large and small length scales.

The pie charts in Figure 2(B) show the water and lipid distributions in the radial direction of the LNPs, deduced following the core–shells model analysis. The LNP radius increased from 467 to 530 Å upon pH shift from neutral to acidic, resulting in an increase in the total LNP volume by 46.6% (Figure 2(C)). The total amount of lipids inside the LNPs remained constant during the pH shift, indicating that the LNPs did not undergo fusion or lysis upon swelling. Thus, the increased LNP volume arose from water ingress during the pH shift; the averaged water fraction increased from 30.9% (pH 7.4) to 53.1% (pH 4). Moreover, the core, mid shell, and inner shell observed at pH 7.4 merge into a uniform core region at pH 4, leaving only the outer shell as a distinct region by its water volume fraction. Consequently, the water volume fraction underwent less fluctuation within LNP under acidic conditions. The fluctuation of water distribution can be evaluated qualitatively by an indicator, SD_water_, which stands for the standard deviation (SD) of water volume fraction in different regions of an LNP from the mean value (further details given in SI 7). The results show that the SD_water_ of the SOPC_pLuc LNPs decreased from 7.0% at pH 7.4 to 3.3% at pH 4. The decrease in SD_water_ value means that water distribution across different regions within LNP became less varied. Although SANS has not tracked the precise location of the plasmid in this study, Brader et al.^51^ used cryo-EM with thionine staining to reveal that the cargo can relocate within mRNA-LNP along with pH decrease.

The mechanism of this LNP swelling and component redistribution is analogous to the findings in other pH-sensitive systems of polymers^52^ and lipids.^53^ At pH 7.4, the LNP internal nanostructure resulted from the compromised balance between the elasticity of the plasmid, the packing of lipids, and the interactions among the lipid headgroups, counterions, plasmids, and water molecules.^46^ When the pH decreased to 4, the MC3 molecules in LNP were predominantly protonated (p*K*_a_ of MC3 is ∼6.4^5,28^), and there were insufficient nucleic acids to match the charges (due to the N/P ratio being 6). The increased electrostatic repulsion between the charged MC3 lipids surpasses the attraction between the charged MC3 lipids and oppositely charged nucleic acid encapsulated in the LNP and then triggered the LNP’s AISE as the dominant factor of the LNP structure. During the AISE, the excess charges of MC3 lipids were eventually balanced by counterion binding. Water also follows the inward flow of ions due to the osmotic pressure, leading the LNP swelling.

In agreement with the SANS analysis, the increased hydrodynamic radius and stable PDI upon pH shift evince an expansion of the LNPs during AISE (without particle aggregation, fusion, or lysis), accompanied by a switch in zeta potential from negative to positive when the pH decreased to 4. It implies the presence of MC3 head groups at the LNP surface because MC3 is the only positively charged lipid at pH 4. A similar zeta potential switch phenomenon has been reported in an LNP-mRNA system.^54^ The zeta potential switch can enhance the electrostatic attraction between the net positive LNPs and the negatively charged endosomal membranes, promoting the endosome escape.^55^

The T–S model parameters in Table 1 indicate that the peak features (at the *q* value around 0.1 Å^–1^) at both pH 7.4 and 4 refer to a similar nanostructure. However, Figure 2(A) shows the peak feature becomes more pronounced at pH 4, suggesting the interface between water and lipid domains expanded during AISE, well-matched with the increased averaged water volume fraction from the core–shell model analysis.

### Effects of Payloads on LNP Structure, AISE, and Function

**Figure 3:**
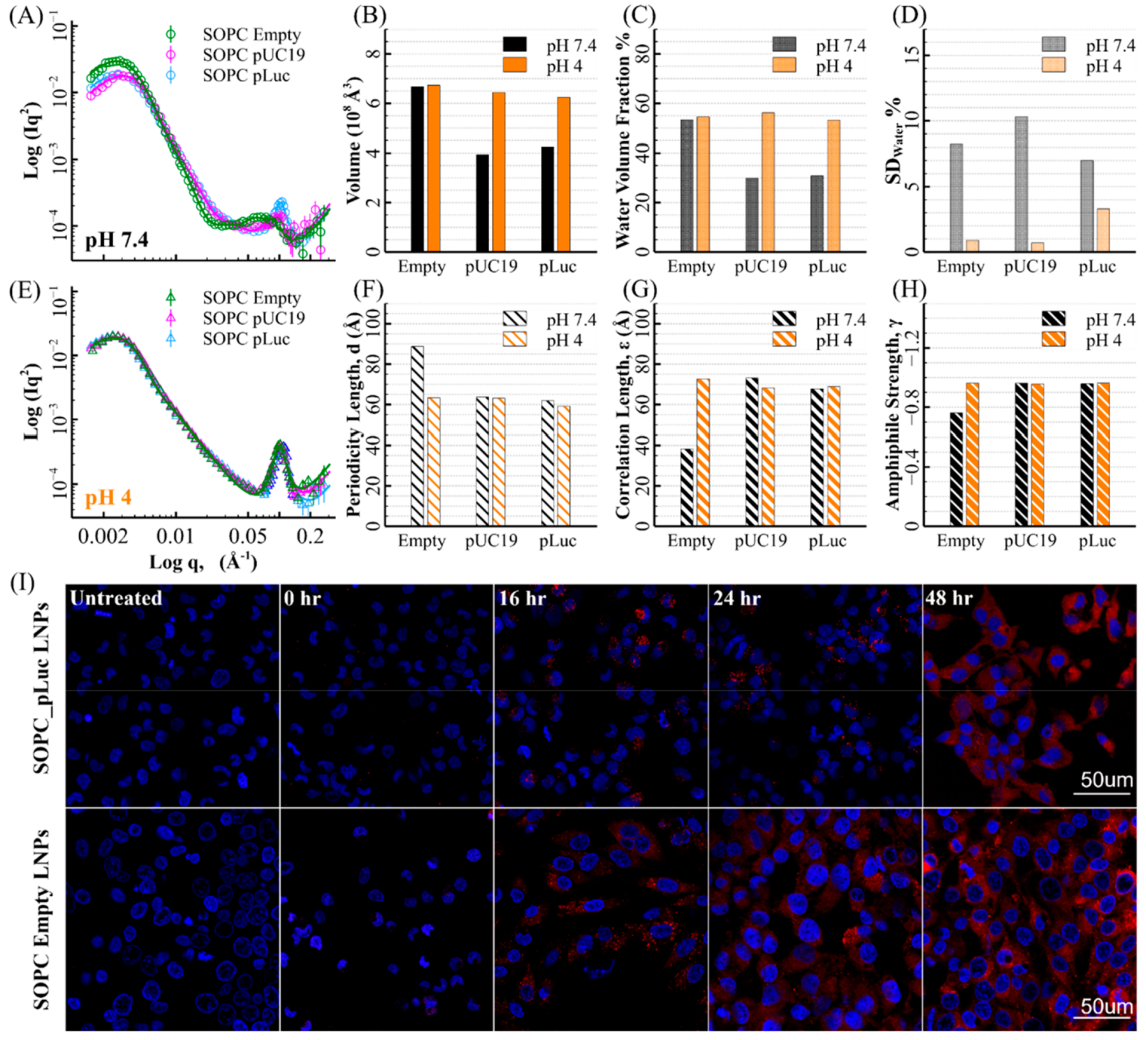
Effects of payloads on LNP structure and function. (A–E) SANS profiles of the SOPC LNPs encapsulating plasmids pUC19 (magenta) and pLuc (blue) as payloads and no payload (green) at pH 7.4 (A) and pH 4 (E), with continuous lines as the best fits. The core–shells model’s parameters, LNP’s total volume, averaged water volume fraction, and SD_water_ are plotted in the bar charts (B–D). The T–S model’s parameters, periodicity length *d*, correlation length ε, and amphiphile strength γ are plotted in the bar charts (F–H). (I) DID tracer imaging experiment of SOPC LNPs with and without payloads. Confocal images were taken from Hela cells with nuclei stained by DAPI in blue and LNPs labeled with fluorescent lipophilic tracer DID showing red color upon release at 0, 16, 24, and 48 h after transfection with LNPs at the concentration of 50 ng/well.

In order to examine the effects of different sized payloads on the LNP nanostructure changes during AISE, three SOPC LNP formulations were manufactured using the same FNC method: (i) no payload (empty LNPs), (ii) pUC19 (2.6k bps) payload, and (iii) pLuc (5.5k bps) payload. Figure 3(A) and (E) show the plots of the measured and best-fitted SANS profiles. The best-fit parameters are presented in the bar charts in Figure 3 and listed in SI 6.

The SANS profiles of the SOPC LNPs encapsulating plasmids (pUC19 (magenta) and pLuc (blue)) overlap well at both pH 7.4 (Figure 3(A)) and pH 4 (Figure 3(E)), suggesting the impact of the size of the pDNA payload on the LNP structure was not evident by SANS.

On the other hand, at pH 7.4, the empty SOPC LNP has a less ordered nanostructure than the LNPs with payload, which is shown by the broader peak feature in Figures 3(A) and the fitted T–S model parameters in Figure 3(F)–(H). Also, the empty SOPC LNP has a higher water volume fraction. These differences may arise during the LNP formation process, in which the electrostatic attraction between the charged MC3 lipid and plasmid plays a critical role in condensing the components. However, the structural differences between the LNPs with and without payload collapsed at pH 4. It indicates that the role of the pDNA payload in determining LNP nanostructure is secondary to the impact of electrostatic repulsion raised by the charges carried by the MC3 molecules.

**Figure 4:**
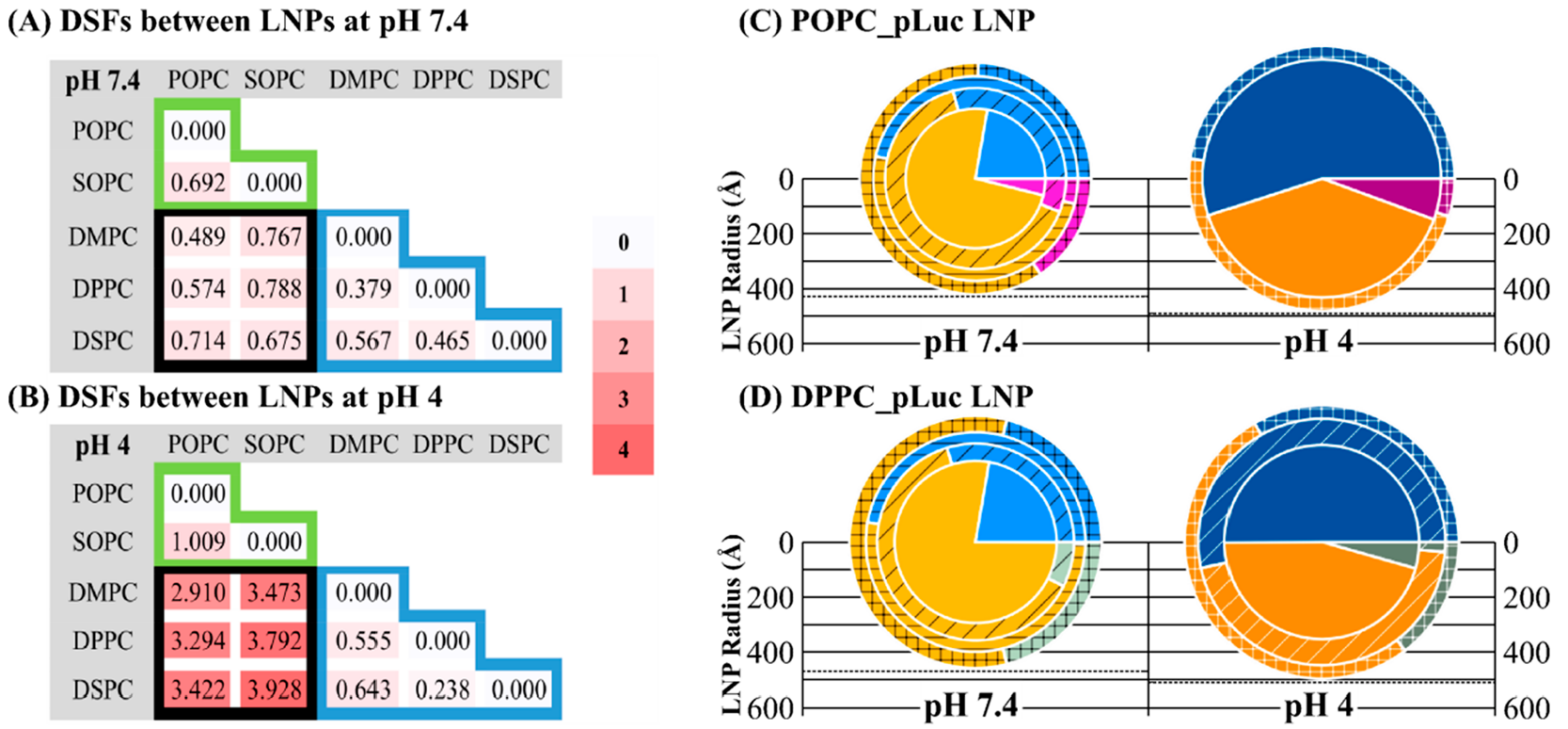
Structural differences between LNPs with different helper lipids. (A, B) The DSFs between pairs of LNPs at pH 7.4 (A) and pH 4 (B). The sequential color scale was used to help visualize the values. DSF equals 0 when an LNP is compared with itself. The green border highlights the LNP pairs within the unsaturated helper lipid group (SOPC, POPC). The blue border encloses the LNP pairs within the saturated helper lipid group (DMPC, DPPC, DSPC). The black border covers the LNP pairs consisting of one saturated and one unsaturated helper lipids. (C, D) Lipids and water distributions in POPC_pLuc LNP (C) and DPPC_pLuc LNP (D) at pH 7.4 and pH 4 (labeled). The radii of the pie charts are proportional to the dimensions of the LNPs. Water is labeled blue at pH 7.4 and dark blue at pH 4; POPC is labeled magenta (at pH 7.4) and dark magenta (at pH 4); DPPC is labeled green at pH 7.4 and dark green at pH 4; other lipids, including lipid MC3, DMG-PEG(2000) and cholesterol, are labeled orange at pH 7.4 and dark orange at pH 4.

DID tracer imaging revealed the location of the LNPs and identified any disassembly during cell transfection, indirectly examining the function of the LNPs (DID is an environment-sensitive carbocyanine dye that shows far-red fluorescence when incorporated in lipophilic environments). Confocal images taken at 0, 16, 24, and 48 h after adding LNPs to the Hela cells are shown in Figure 4(I). It is clear that only at 16 h are LNPs internalized and accumulated around the cell nuclei. At 24–48 h, a wider distribution is observed, suggesting the disassembly of the LNPs after their internalization and subsequent release of payload. Dye release was similar between SOPC LNPs with and without plasmid, suggesting they underwent a similar mechanistic path after cellular uptake. It supports the SANS observation from another aspect that the LNPs with and without payload have a similar structure after the acidification.

### Effects of Helper Lipids on LNP Structure and AISE

Four additional phosphatidylcholine helper lipids: 1-palmitoyl-2-oleoyl-*sn*-glycero-3-phosphocholine (POPC), 1,2-distearoyl-*sn*-glycero-3-phosphorylcholine (DSPC), 1,2-dipalmitoyl-*sn*-glycero-3-phosphocholine (DPPC), and 1,2-dimyristoyl-*sn*-glycero-3-phosphocholine (DMPC), were used to substitute for SOPC to test how different tail groups of the helper lipid affect LNP structure and AISE. As shown in Figure S1, DSPC (18:0–18:0), DPPC (16:0–16:0), and DMPC (14:0–14:0) each have two identical saturated fatty acyl chains as their tail groups. SOPC (18:0–18:1) and POPC (16:0–18:1) each have one saturated and one unsaturated fatty acid chain as their tail groups. All the LNPs examined were manufactured to encapsulate pLuc following the same FNC method. The SANS profiles of the LNPs containing different helper lipids were collected at both pH 7.4 and pH 4 and fitted using the combined core–shells and T–S model. The collected and best-fitted SANS profiles are shown in Figure S8, and the key parameters are listed in Tables S5 and S6. The deduced LNP structure parameters, such as LNP total volume, lipid/water volume fractions, and lipid/water volumes of different LNP regions, were calculated from the best-fitted parameters, shown in Figures S7–S10.

Because 15 structural parameters are required to describe the structural features of an LNP, a structural feature space (LSF) was generated to convert all the structural information obtained from the experimental data into a single feature vector space, where different LNPs’ structures could be compared just by their positions. As explained in SI 8, each LNP has a unique position in the LSF consisting of 15 numerical elements matching the 15 structure parameters. The Euclidean distance in the structure feature space (DSF) between two LNPs in LSF has been used to evaluate their similarity. DSF is a non-negative real number and is equal to 0 when an LNP is compared with itself; a larger DSF indicates less similarity. Figure 4(A) and (B) show the DSF values between each pair of LNPs at pH 7.4 and pH 4. These DSF values indicate that the LNPs’ structures can be classified into two groups by the type of helper lipids, i.e., unsaturated and saturated. The values and corresponding color scales in Figure 4(A) reveal rather small differences between all the LNPs at pH 7.4, even between LNPs from the two different helper lipid groups. In contrast, the area with a black border in Figure 4(B) indicates that the unsaturated helper lipid group is distinct from the saturated helper lipid group at pH 4, while the difference within each group (showed by the area with a green or blue border) remains relatively small.

Figure 4(C) and (D) show the lipid and water distributions of POPC_pLuc LNPs and DPPC_pLuc LNPs representing the LNPs containing unsaturated and saturated helper lipids, respectively. With the help of partially deuterated lipids, D62-DPPC and D31-POPC, it was possible to distinguish the helper lipids from the other lipid components (MC3, PEG lipid, and cholesterol) from SANS measurements, enabling their distributions in each region of the LNP to be determined. Further details regarding the experiments using deuterated lipids are given in SI 6.

At pH 7.4, both POPC_pLuc LNPs and DPPC_pLuc LNP have a similar core–shells structure and water distribution to the SOPC_pLuc LNPs as discussed previously. Interestingly, both POPC and DPPC helper lipids became preferentially located in the LNP outer shell at pH 7.4, analogous to the finding reported by Arteta^22^ in an mRNA-LNP system using DSPC as the helper lipid. Also, it is in line with a structure model proposed using NMR by Viger-Gravel et al.,^34^ in which the LNP has a shell enriched with PEG and helper lipid and a core mainly consists of cholesterol and ionizable lipid. This localization of helper lipids could be attributed to their cylindrical molecular shape (adopting the spontaneous curvature close to 0), promoting lipid bilayer phases.^16,56^ Moreover, in contrast to the absence of DPPC in the core region, 13% POPC can be present in this region rich with MC3 lipid and cholesterol. It implies that the better flexibility of the unsaturated tail may promote the solubility or packing of helper lipid in MC3 and cholesterol.

During the AISE, the helper lipid volume fraction in the out shell of POPC_pLuc LNP was dramatically reduced from 15.5% to 4.6%. The POPC was redistributed to the inner regions of the LNP, resulting in a more homogeneous distribution of the helper lipid across the LNP at pH 4. Accompanying the helper lipid redistribution, the LNP was swelling by water ingress and the water distribution within the LNP also became more homogeneous at pH 4. This redistribution of water and helper lipid may contribute to the modulation of charge density in the LNP inner region raised by the MC3 molecules.^57^ Because helper lipid plays a critical role in supporting bilayer structure formation, redistribution of the helper lipid out from the outer shell can decrease the spontaneous curvature of the remaining lipid mixture, promoting a nonbilayer structure formation. The water volume fraction in the outer shell almost doubled from 25% to 48%, also implying the formation of nonbilayer structure or defects in the LNP outer membrane. It has been reported that the nonbilayer structure in the LNP outer membrane facilitates the fusion of the LNP with the endosomal membrane, promoting endosome escape.^8,58^ Also, Arteta et al.^22^ showed that a high density of DSPC helper lipid on the LNP surface reduced the protein expression. Moreover, PEGylated lipid distributes on the outer surface of the LNP and can stabilize LNPs *in vivo* but hamper their endosomal release.^42,57,59−61^ The redistribution of components in the LNP outer membrane during the AISE may help overcome this.

DPPC_pLuc LNP had a less extent of helper lipid redistribution during the AISE. Only 12% of helper lipid in the DPPC_pLuc LNP was redistributed out from the outer shell, leaving the LNP outer membrane still a helper lipid-rich region containing 66% of the DPPC in the LNP. The hydration increase in the outer shell of DPPC LNP is also lower than the POPC LNP during AISE. At pH 4, the water volume fraction in the outer shell was 33% for DPPC LNP against 48% for POPC LNP. As a result, DPPC_pLuc LNPs had less water ingress and total volume increase during pH decreases, and the SD_water_ values at pH 4 (8.4% for DPPC LNPs against 3.3% for POPC LNPs) show that the water distribution within DPPC_pLuc LNPs is less homogeneous.

The best-fitted parameters from the SANS analysis of the LNPs containing other help lipids (DSPC, SOPC, DMPC) are listed in SI 6. In summary, both saturated and unsaturated lipids are preferentially located in the LNP outer shell at pH 7.4, supporting the lipid bilayer structure as the LNP outer membrane. At pH 4, due to the protonated MC3 head groups, there was a trend of LNP internal structural rearrangement that required helper lipid redistribution. However, in this process, unsaturated helper lipids showed a better ability to relocate, resulting in a greater extent of AISE. As a result, the structural differences between LNPs at pH 7.4 and 4 became more significant.

Table 2 shows the best-fitted parameters in the T–S model, depicting the nanostructure features of the POPC and DPPC LNPs on the small length scale. Despite the peak feature at *q* position about 0.1 Å^–1^ becoming more pronounced at pH 4 for both LNPs, the ratios of the parameters of POPC_pLuc LNPs from the two pH values are close to 1, indicating little variation in its nanostructure type during the pH shift. DPPC LNPs have the same nanostructure as POPC LNPs at pH 4 but a slightly different nanostructure at pH 7.4. This difference at pH 7.4 may be due to the helper lipid’s absence in the DPPC_pLuc LNP core and midshell.

**Table 2:** T–S Model Parameters of POPC_pLuc LNP and DPPC_pLuc LNP at pH 7.4 and 4

	POPC_pLuc LNPs			DPPC_pLuc LNPs		
pH	*d*(±0.5 Å)	ε (±0.5 Å)	γ (±2%)	*d*(±0.5 Å)	ε (±0.5 Å)	γ (±2%)
pH 7.4	63.2	66.8	–0.956	66.7	48.6	–0.909
pH 4	58.0	72.7	–0.968	58.7	71.3	–0.966
ratio^a^	0.92	1.09	1.01	0.88	1.47	1.06

a The ratio of the parameters at pH 4 and 7.4.

### *In Vitro* Expression Efficiency Correlates with the Extent of LNP AISE

**Figure 5:**
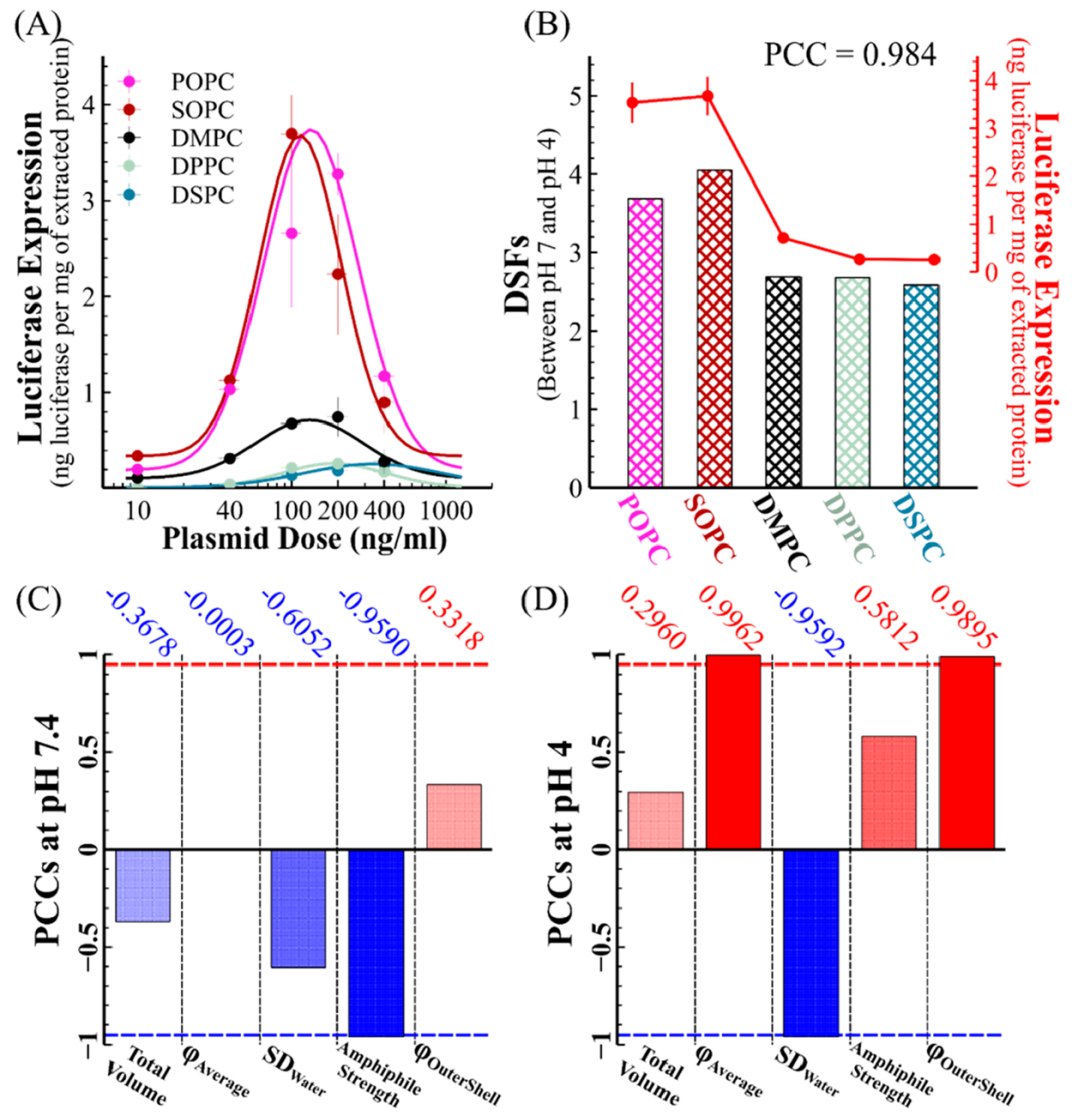
*In vitro* luciferase expression correlates with the extent of LNP AISE. (A) *In vitro* luciferase expression following incubation of cultured Hela cells with LNPs containing different helper lipids. Plasmid dose affects the luciferase expression of LNPs containing different helper lipids. (B) The bar chart shows the DSF value as the distance between the LSF positions of each LNP at pH 7.4 and 4. The scatter plot (red) shows the optimal luciferase expressions (the right *y*-axis) shown in (A). (C, D) The bar charts show the PCC (Pearson correlation coefficient) values of the correlation between the optimal Luciferase expressions and the key LNP structural parameters at pH 7.4 (C) and pH 4 (D). φ = averaged water volume fraction. The PCC values are also shown above each bar. The dashed lines show the PPC values equal to 0.95 (red) and −0.95 (blue), beyond which the correlation can be regarded as a strong linear correlation.

Figure 5(A) shows the *in vitro* luciferase expression following incubation of Hela cells with LNPs incorporating different helper lipids. Despite different expression levels, luciferase expression for all LNPs shows a distinct bell-shaped distribution against plasmid concentration. The results show that the helper lipid type can dramatically affect LNP function, even though its molar ratio in the formulation is only 10%. The optimal *luc* expressions are shown by scatters in Figure 5(B) and follow the order SOPC ≈ POPC ≫ DMPC > DPPC ≈ DSPC, with the helper lipids containing unsaturated tails displaying far greater expressions (max 15-fold higher) than those with saturated tails. The trend that POPC and SOPC enhance protein expression over DSPC in Hela cells is in agreement with Cullis et al.,^15^ who showed that unsaturated lipids bearing phosphatidyl moieties promote protein expressions for several cell lines.

A key finding in this work is the correlation between the LNPs’ *in vitro* expression and the extent of AISE, shown in Figure 5(B). The Pearson correlation coefficient (PCC) was 0.984, indicating a strong positive linear correlation (SI 9). The extent of AISE is evaluated by the DSF of each LNP between pH 7.4 and 4. The helper lipids with an unsaturated chain (SOPC and POPC) enhance both protein expression of the gene delivered and AISE. SANS measurements showed that lipids with unsaturated tails were more readily redistributed, participating in the evolution of the LNP structure during pH decrease. Consequently, unsaturated helper lipids can promote the redistribution of other LNP constituents during AISE and lead to a better payload release.

Figure 5(C) and (D) show the correlation between luciferase expression and individual LNP nanostructure features. There was a weak correlation between the total LNP volume and cell transfection at each pH, indicating that the LNP size was not the key determinant of the function of LNPs with different helper lipids. At pH 4, *luc* expression strongly correlated with the LNP averaged water volume fraction and SD_water_, revealing the role played by the higher level of hydration and the more homogeneous water distribution of LNP in an acidic environment. Moreover, amphiphile strength is the only parameter strongly correlated with expression at pH 7.4. This correlation becomes weaker at pH 4 because the difference between the observed nanostructures between LNPs vanishes at pH 4. Notably, the positive correlation between expression and the outer shell’s water volume fraction is much stronger at pH 4 than at pH 7.4, consistent with the observation that an increase in the hydration of the outer shell during the AISE generally enhances LNP function.

## Conclusion

This study of LNP AISE provides a sound structural basis to answer the question of the effect of redistribution of helper and ionizable lipids on the LNP’s function as a vector for the delivery of genetic material. The SANS data revealed that AISE results from (i) protonation of the ionizable lipid, driving LNP nanostructure rearrangement that is associated with increased water mobility and lipid redistribution; (ii) migration of the helper lipid (especially unsaturated) from the outer shell toward the core can drive the observed change in the LNP outer membrane such as increased water volume fraction, surface zeta potential switch, and can potentially lead to reducing PEGylation of LNP surface and nonbilayer structure formation, all of which promote the interaction between LNP and endosome membrane; (iii) and the appearance of unsaturated helper lipids to have a better mixing ability with the MC3 lipid and cholesterol and increased mobility within the LNP than saturated helper lipids, leading the LNP to a larger extent of AISE and a higher expression of luciferase. Instead of focusing on the LNP nanostructure at physiological pH, these data demonstrate the importance of examining the formulation–structure–function relationship over a pH shift to provide better mimicry of endosomal maturation. This study offers a potential strategy during early stage development for optimizing LNP components and formulation to increase protein production.

## Materials and Methods

### Chemicals

The ionizable lipid *O*-(*Z*,*Z*,*Z*,*Z*-heptatriaconta-6,9,26,29-tetraem-19-yl)-4-(*N*,*N*-dimethylamino)butanoate (DL in-MC3-DMA) was synthesized at the ISIS Deuteration Facility, Rutherford Appleton Laboratory (RAL), Science and Technology Facilities Council (STFC), UK. DMG-PEG 2000 (1,2-dimyristoyl-*rac*-glycero-3-methoxypolyethylene glycol-2000) was purchased from NOF AMERICA Corporation, US. The helper lipids, including SOPC (1-stearoyl-2-oleoyl-*sn*-glycero-3-phosphocholine), DSPC (1,2-distearoyl-*sn*-glycero-3-phosphocholine), POPC (1-palmitoyl-2-oleoylglycero-3-phosphocholine), DPPC (1,2-dipalmitoyl-*sn*-glycero-3-phosphocholine), and cholesterol, were obtained from Avanti Polar Lipids, US. Deuterated DPPC (1,2-dipalmitoyl-*d*_62_-*sn*-glycero-3-phosphocholine) and POPC (1-palmitoyl-*d*_31_-2-oleoyl-*sn*-glycero-3-phosphocholine) were also purchased from Avanti Polar Lipids. All lipids were used without any further purification. Hydrogenous phosphate-buffered saline (PBS) and citrate buffers were prepared using ultrahigh-quality (UHQ) H_2_O processed from an Elgastat PURELAB purification system. The deuterated buffers were prepared using D_2_O from Sigma-Aldrich. The chemicals for buffer preparation (sodium phosphate dibasic, sodium phosphate monobasic, citric acid, sodium citrate tribasic, sodium chloride) were obtained from Sigma-Aldrich. The plasmids pUC19 and pLuc were provided by AstraZeneca.

### Dynamic Light Scattering (DLS)

The LNP hydrodynamic diameter, size polydispersity, and surface zeta potential were measured using Zetasizer Nano ZS (Malvern Instruments Ltd.).

### Encapsulation Efficiency

The encapsulation efficiency and plasmid concentration of all LNP samples were measured using Quant-iT PicoGreen dsDNA Assay. The measurements were performed on a Fluorolog-3 Spectrofluorometer (HORIBA).

### Cryo-TEM

Cryo-TEM measurements were performed on Talos Arctica (200 kV) with a Falcon 3 linear detector at the Cryo-EM Facility, Department of Biochemistry, University of Cambridge, UK. A 3 μL, 20 mg/mL (lipid concentration) LNP sample was applied to a perforated carbon grid. The grid was glow discharged for 60 s before the experiment. The excess sample was removed from the grid by blotting with filter paper. Then, the grid was plunge-frozen in liquid ethane using a Vitrobot System developed by Thermo Fisher Scientific.

### Small-Angle Neutron Scattering (SANS)

SANS measurements were performed using the instrument SANS2D at ISIS Neutron Facility, RAL, STFC, UK. SANS scattering intensity, *I(q)*, was measured as a function of the momentum transfer (*q*), , where λ is the incident neutron wavelength and 2θ is the scattering angle. LNP samples were prepared at the concentration of 2.5 mg/mL and filled in Hellma quartz banjo-shaped cuvettes. A water bath system was used to keep the samples at 25 °C. The data used for analysis was obtained from the original data through a standard data reduction process, which used the measurements of solvent, empty cell and transmission for correction. SANS data analysis was performed using SASView (version.5.0.3, http://www.sasview.org), and the more detailed analysis processes are described in SI 5 and SI 6.

### Cell Culture

Hela cells were maintained in Dulbecco’s Modified Eagle’s Medium (DMEM) supplemented with 10% fetal bovine serum (FBS), 100 U mL^–1^ penicillin, and 100 μg mL^–1^ streptomycin and incubated at 37 °C in an atmosphere of 5% CO_2_.

### Transfection

For transfection experiments, Hela cells were seeded at a density of 4 × 10^4^ cells per well in 24-well plates and allowed to adhere for 24 h. LNP suspensions or lipofectamine-plasmid complexes of varying concentrations of DNA dose per well were added to the cells. After the cells were incubated for 48 h, media were removed, and the cells were carefully washed 1× with PBS (pH 7.4). For lipofectamine controls, 4 μL of lipofectamine 2000 transfection reagent (Invitrogen) was added to varying concentrations of plasmid (0.5–5000 ng) per well in serum-free media to create lipofectamine–plasmid complexes. Lipofectamine–plasmid complexes were added to cells and incubated in serum-free conditions for 6 h and then replaced with fresh media (with or without serum according to experimental design).

### Luciferase Assay

One hundred microliters of reporter lysis buffer (Promega UK) was added to each well, and Hela cells were subjected to two freeze–thaw cycles. Twenty microliters of cell lysate from each well were assayed using a luciferase assay kit (Promega) and read immediately on a luminometer (Biotek Synergy H1). The luciferase activity was converted to the amount of luciferase expressed using a recombinant luciferase protein (Promega) as the standard. Total protein content in the lysate was measured by DC Protein Assay (Bio-Rad), and luciferase expressed was normalized against total protein content.

### Cell Viability Confocal Imaging

One hundred microliters of Hoechst 33342 (5 ng/mL) and 100 μL of propidium iodide (1 ng/mL) were added to PBS-washed Hela cells and incubated for 20 min. Cells were then washed 3× with PBS (pH 7.4) and immediately observed using a laser scanning confocal microscope (Leica SP8 Inverted). Confocal images were then analyzed using ImageJ (version 1.47) using the mean fluorescence intensity of the image taken in each channel to calculate the percentage of cell death.

### DID Tracer Imaging

0.2% (molar) 1,1′-dioctadecyl-3,3,3′,3′-tetramethylindodicarbocyanine, 4-chlorobenzenesulfonate (DID) was added to the LNPs, with noncapsulated DID molecules removed by dialysis. Hela cells were transfected with LNPs as previously described and incubated between 6 and 24 h. At this point, cells were washed 1× with PBS (pH 7.4) and incubated with 100 μL of Hoechst 33342 (5 ng/mL) for 20 min. Cells were then washed 3× with PBS (pH 7.4) and immediately observed using a laser scanning confocal microscope (Leica SP8 Inverted).

## Supporting Information Available

Molecular structures of the components of LNPs, LNP manufacturing device setup, LNP manufacturing procedure and procedure optimization, additional Cryo-TEM images, additional luciferase expression and cytotoxicity data, further explanation of SANS data analysis, SANS data and best-fit simulation profiles and parameters, SD_water_ values, LNP’s structural feature space, similarity analysis, and correlation coefficient (PDF)
