# Supplementary material for: Acidification-Induced Structure Evolution of Lipid Nanoparticles Correlates with Their In Vitro Gene Transfections
Source: ACS Nano. 2023 Jan 6;17(2):979–90. doi: 10.1021/acsnano.2c06213 (PMC9878718; doi:10.1021/acsnano.2c06213)
Supplement: Supplementary file 1 — nn2c06213_si_001.pdf [file nn2c06213_si_001.pdf]

Supplementary Information for

Acidification-Induced Structure Evolution of Lipid Nanoparticles Correlates with Their *in Vitro* Gene Transfections

Zongyi Li[1], Jessica Carter[1], Luis Santos[2], Carl Webster[3], Christopher F. van der Walle[4], Peixun Li[5], Sarah E. Rogers[5], Jian Ren Lu[1*]

[1]Biological Physics Laboratory, School of Physics and Astronomy, University of Manchester, Oxford Road, Schuster Building, Manchester M13 9PL, UK.

[2]Dosage Form Design Development, Biopharmaceuticals Development, AstraZeneca, Gaithersburg, MD 20878, USA.

[3]Discovery Sciences, R&D, AstraZeneca, Cambridge CB21 6GH, UK.

[4]The Cell and Gene Therapy Catapult, The Centre for Regenerative Medicine, 5 Little France Drive, Edinburgh EH16 4UU, UK.

[5]ISIS Neutron Facility, STFC, Chilton, Didcot OX11 0QZ, UK.

*Corresponding author: Jian R Lu

Email: j.lu@manchester.ac.uk; Tel: +44 161 2003926

SI 1. Molecular Structures of the Lipid Components in the LNP Systems

Each LNP system contained four lipid components: MC3, cholesterol, DMG-PEG(2000) and SOPC (or other helper lipid). Their structures together with four other helper lipids are shown below.

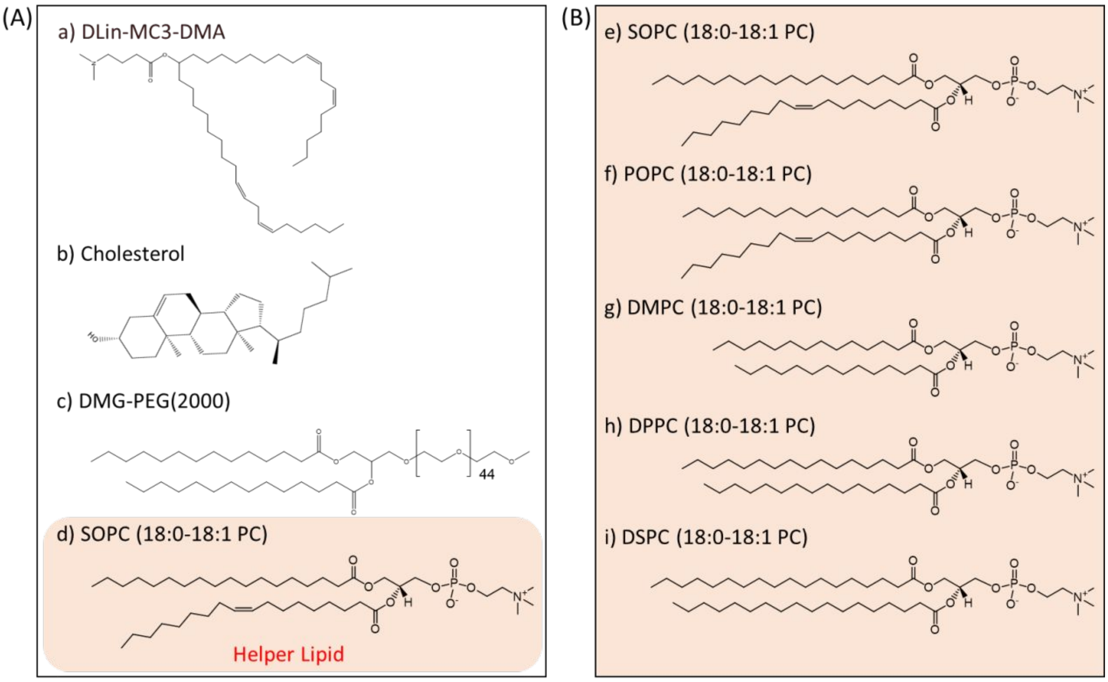

Figure S1. The molecular structures of (A) the four lipid components in the LNP formulation, and (B) the alternative helper lipids: POPC, DMPC, DPPC, DSPC.

SI 2. LNP Preparation

SI 2.1 The CIJ Device and LNP Manufacturing Process

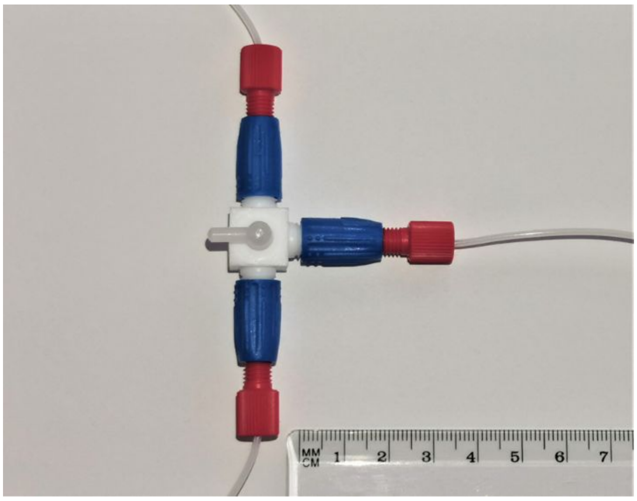

Figure S2. A photo of the CIJ device based on a T-junction mixer.

LNPs were prepared by the flash nano-complexation (FNC) method using a confined impinging jet (CIJ) based on a T-junction mixer, as shown in **Figure S2**. The PTFE T-junction mixer, syringe adaptors and PTFE tubing were purchased from Cole-Parmer Instrument Company Ltd, UK. The T-junction mixer has an inner diameter of 1.5 mm, and the tubing has an inner diameter of 0.5 mm. The plasmid and lipid mixture solutions were loaded into 20 ml Terumo plastic disposable syringes, and operated by a programmable syringe pump (CHEMYX, model FUSION-4000). The output stream from the T-junction mixer was collected in a beaker after passing through a PTFE tubing with a length

of 12 cm. The first 1 ml and the last 0.5 ml of the LNP suspension were discarded to ensure that the collected LNPs were formed at a stabilized mixing condition.

The collected LNP suspension was dialyzed against a 10 mM PBS buffer (138 mM NaCl, pH 7.4) using a dialysis tubing cellulose membrane purchased from Sigma-Aldrich. The molecular weight cut-off of the membrane was 14 kDa. After dialysis, the collected sample was concentrated by centrifugation (4000x g) using Amicon Centrifugal Filter Unit (10 kDa).

SI 2.2 Optimization of the Manufacturing Parameters

The flow rates of water and ethanol streams had been optimized through a screening process to obtain the LNPs with a monodispersed size distribution (PDI < 0.1) and best encapsulation efficiency (> 90%).

The screening process was first performed for SOPC LNPs encapsulating the plasmid pLuc. Six flow rate combinations were chosen by two output flow rates and three flow rate ratios between the water and ethanol streams (6 flow rate combinations = 2 output flow rates × 3 flow rate ratios). The output flow rate and the sum of the two input flow rates were tested at 5 ml/min and 10 ml/min, and the input flow rate ratios were tested at 3:1, 3:2 and 3:3 (water stream: ethanol stream).

The plasmid concentration remained fixed at 0.01 mg/ml in all the tests. In contrast, the concentration of the lipid MC3 (w/v) was calculated using the formula below to ensure the N/P ratio remained at 6:

$$C_{MC3} = C_{Plasmid} \cdot \frac{v_{Plasmid}}{v_{Lipids}} \cdot \frac{Q_{plasmid}}{MW_{plasmid}} \cdot R_{N/P} \cdot MW_{MC3} \qquad S(1)$$

where C_{MC3} is the concentration (w/v) of MC3 in the lipid mixture solution; $C_{Plasmid}$ is the concentration (w/v) of plasmid, which remained constant at 0.01mg/ml; $υ_{Plasmid}$ and $υ_{lipids}$ are the flow rates of the plasmid and lipid mixture solutions, respectively; $R_{N/P}$ is the N/P ratio, which was constant at 6; $Q_{Plasmid}$ and $MW_{Plasmid}$ are the molar charge and molar weight of the plasmid; MW_{MC3} is the molar weight of MC3. The molar ratio of MC3: cholesterol: SOPC: DMG-PEG (2000) was kept at 50:38.5:10:1.5.

The diameter, PDI and the encapsulation efficiency of the six batches of LNPs were measured by DLS and PicoGreen™ dsDNA assay using a Fluorolog-3 Spectrofluorometer (HORIBA). The results are shown in Figure S3 and **Table S1**. From Figure S3 (A), the optimized combination of flow rates for the SOPC_pLuc LNPs is 6 ml/min for the water stream and 4 ml/min for the ethanol stream, resulting in an output flow rate of 10 ml/min.

This work involved LNPs with two plasmids and two helper lipids, and the screening processes covered SOPC_pUC19 LNPs, DSPC_pLuc LNPs and DSPC_pUC19 LNPs. The rest of the data are shown in **Figure S3** (B) (C) and (D), respectively. Under the optimized conditions, differences between helper lipids or plasmids were small. Thus, the optimized flow rate combination (water streams: ethanol streams = 6 ml/min: 4 ml/min) was used to produce all the LNPs used in this work. The Reynolds number (Re) of the CIJ device was calculated following the formula[1]

$$Re = \frac{4}{\pi d_w} \cdot \frac{\rho_w Q_w}{\mu_w} + \frac{4}{\pi d_e} \cdot \frac{\rho_e Q_e}{\mu_e} \qquad S(2)$$

where d is the inlet diameter of the stream (m); ρ is the density of the fluid in the stream (kgm^{-3}); Q_w and Q_e are the inlet water and ethanol steam flow rates (m^3s^{-1}), respectively; μ is the viscosity of the fluid (Pa·s). For the device used in this work, Re = 125.5 at the optimized flow rate combination.

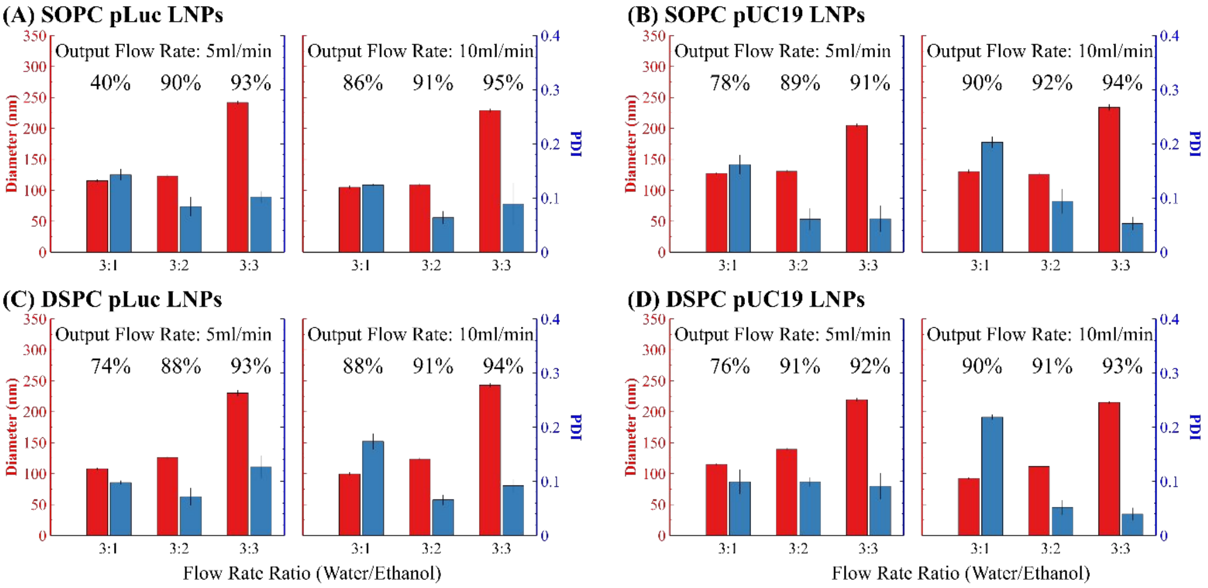

Figure S3. Flow rate and flow rate ratio screening optimize the size, PDI and encapsulation efficiency of the SOPC and DSPC LNPs encapsulating plasmids pLuc and pUC19. The bars represent the results measured by DLS, showing the average diameter of LNPs (red bar and left y-axis) and the diameter PDI (blue bar and right y-axis). The plasmid DNA encapsulation efficiency at each flow rate combination is shown in percentage.

Manufacture Parameters									LNPs' Properties		
Helper Lipid	Plasmid	Total Flow Rate (ml/min)	W/E Flow Rate Ratio	Water Flow Rate (ml/min)	Ethanol Flow Rate (ml/min)	N/P Ratio	Plasmid Conc (mg/ml)	MC3 Lipid Conc (mg/ml)	Diameter (nm)	Diameter PDI	Encapsulation Efficiency
SOPC	pLuc	5	3:1	3.75	1.25	6	0.01	0.374	115.5±1.5	0.143±0.010	40±2%
			3:2	3.00	2.00	6	0.01	0.187	123.4±1.7	0.084±0.017	90±2%
			3:3	2.50	2.50	6	0.01	0.125	242.2±2.4	0.102±0.010	93±2%
		10	3:1	3.75	1.25	6	0.01	0.374	105.2±2.0	0.124±0.001	86±2%
			3:2	3.00	2.00	6	0.01	0.187	109.1±1.6	0.064±0.011	91±2%
			3:3	2.50	2.50	6	0.01	0.125	229.0±2.2	0.089±0.038	95±2%
	pUC19	5	3:1	3.75	1.25	6	0.01	0.374	127.3±0.6	0.162±0.017	78±2%
			3:2	3.00	2.00	6	0.01	0.187	130.8±1.6	0.061±0.019	89±2%
			3:3	2.50	2.50	6	0.01	0.125	205.2±2.5	0.062±0.024	91±2%
		10	3:1	3.75	1.25	6	0.01	0.374	130.5±2.8	0.203±0.010	90±2%
			3:2	3.00	2.00	6	0.01	0.187	126.3±1.5	0.094±0.022	92±2%
			3:3	2.50	2.50	6	0.01	0.125	234.0±5.0	0.053±0.012	94±2%
DSPC	pLuc	5	3:1	3.75	1.25	6	0.01	0.374	107.8±1.3	0.098±0.003	74±2%
			3:2	3.00	2.00	6	0.01	0.187	126.1±1.5	0.072±0.016	88±2%
			3:3	2.50	2.50	6	0.01	0.125	230.3±4.1	0.127±0.020	93±2%
		10	3:1	3.75	1.25	6	0.01	0.374	99.84±1.9	0.174±0.014	88±2%
			3:2	3.00	2.00	6	0.01	0.187	123.9±1.2	0.066±0.009	91±2%
			3:3	2.50	2.50	6	0.01	0.125	243.0±2.7	0.092±0.012	94±2%
	pUC19	5	3:1	3.75	1.25	6	0.01	0.374	115.3±0.8	0.099±0.022	76±2%
			3:2	3.00	2.00	6	0.01	0.187	139.5±1.3	0.099±0.008	91±2%
			3:3	2.50	2.50	6	0.01	0.125	219.7±2.3	0.091±0.024	92±2%
		10	3:1	3.75	1.25	6	0.01	0.374	92.4±0.7	0.219±0.004	90±2%
			3:2	3.00	2.00	6	0.01	0.187	111.6±0.6	0.052±0.013	91±2%
			3:3	2.50	2.50	6	0.01	0.125	215.0±1.4	0.040±0.011	93±2%

Table S1. Screening of the manufacturing parameters (diameter, diameter PDI and the encapsulation efficiency) for the LNPs with two types of helper lipids (DSPC and SOPC) and two plasmids (pLuc and pUC-19). Two optimized combinations of flow rates are shown in red.

Manufacture Parameters of NanoAssemblr Benchtop									LNPs' Properties		
Helper Lipid	Plasmid	Total Flow Rate (ml/min)	W/E Flow Rate Ratio	Water Flow Rate (ml/min)	Ethanol Flow Rate (ml/min)	N/P Ratio	Plasmid Conc (mg/ml)	MC3 Lipid Conc (mg/ml)	Diameter (nm)	Diameter PDI	Encapsulation Efficiency
DSPC	pUC19	12	3:1	9.00	3.00	6	0.107	4.002	104.6±1.0	0.141±0.018	94±2%
			3:1	9.00	3.00	6	0.01	0.374	90.3±1.7	0.138±0.016	85±2%
			3:1	9.00	3.00	9	0.107	6.003	95.3±1.9	0.171±0.008	96±2%
			3:3	6.00	6.00	6	0.01	0.125	182.9±2.7	0.055±0.021	91±2%
	pLuc	12	3:1	9.00	3.00	6	0.107	4.002	80.7±3.1	0.180±0.014	93±2%
SOPC	pLuc	12	3:1	9.00	3.00	6	0.107	4.002	75.7±3.1	0.125±0.010	95±2%
			3:1	9.00	3.00	9	0.107	6.003	73.1±1.5	0.16±0.016	93±2%

Table S2. The manufacturing parameters (diameter, PDI and the encapsulation rate) used in the production of the LNPs by NanoAssemblr Benchtop (UK).

SI 2.3 LNP Preparation for *in vitro* Experiments

The LNP samples used for the *in vitro* experiments were prepared following the method described in Section SI 2.1. Before the sample was concentrated by centrifuging, the LNP samples were sterilized by filtration through a Minisart NML syringe filter (200 nm, Sartorius, UK). The concentrated LNP samples were then diluted into cell culture media to obtain the required concentrations before adding to the cells. All the LNP samples were manufactured within one week before the measurements.

SI 2.4 Buffer Change of the LNP Samples

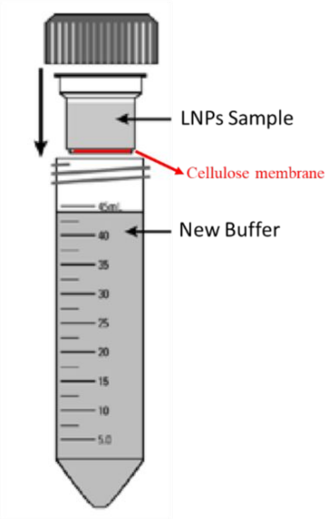

Figure S4. A schematic shows the mini dialysis device Slide-A-Lyzer™ 20K MWCO

The pH or the scattering length density (SLD) of LNP samples in H_2O or D_2O buffer can be changed using a mini dialysis device (Slide-A-Lyzer™ 20 kDa MWCO), as schematically shown in **Figure S4**.

The conical tube was filled with 50 ml new buffer with the required pH, and SLD. 1 ml LNP sample was added to the device (containing a cellulose membrane). Then, the device was placed into the conical tube. The membrane of the device contacted the new buffer. The conical tube was then capped and shaken gently on an orbital shaker. The dialysis buffer was changed 3x at the 2nd, 6th and 12th hours, to ensure a good buffer exchange.

SI 3. Additional Cryo-TEM Images

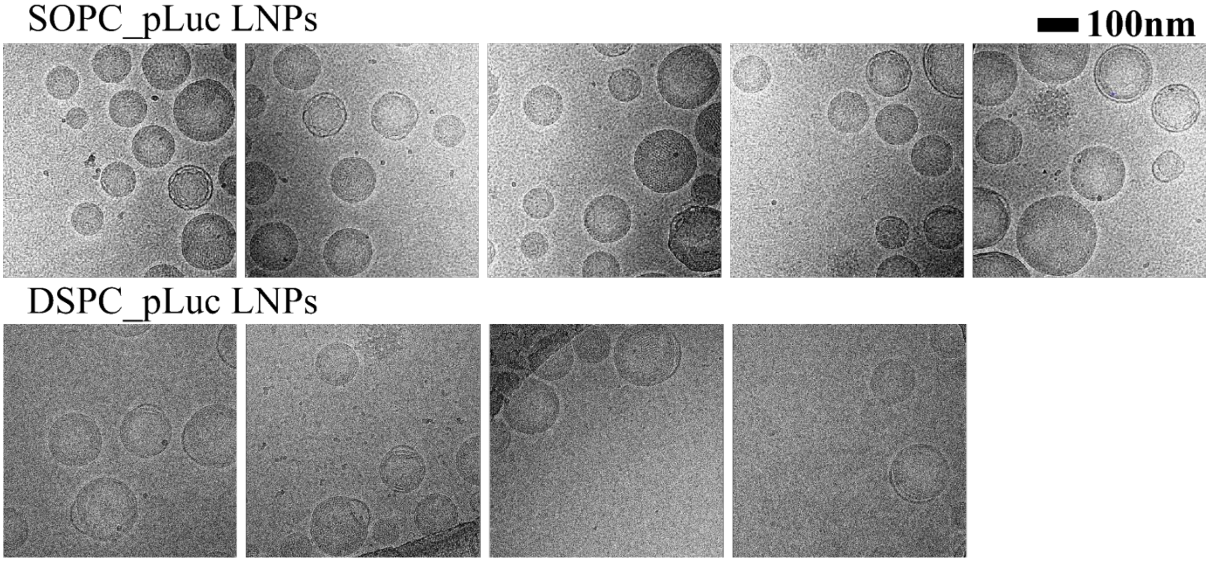

Figure S5. Cryo-TEM images of the SOPC_pLuc LNPs (TOP) and DSPC_pLuc LNPs (Bottom).

SI 4. Luciferase Expression Efficiency and Cytotoxicity

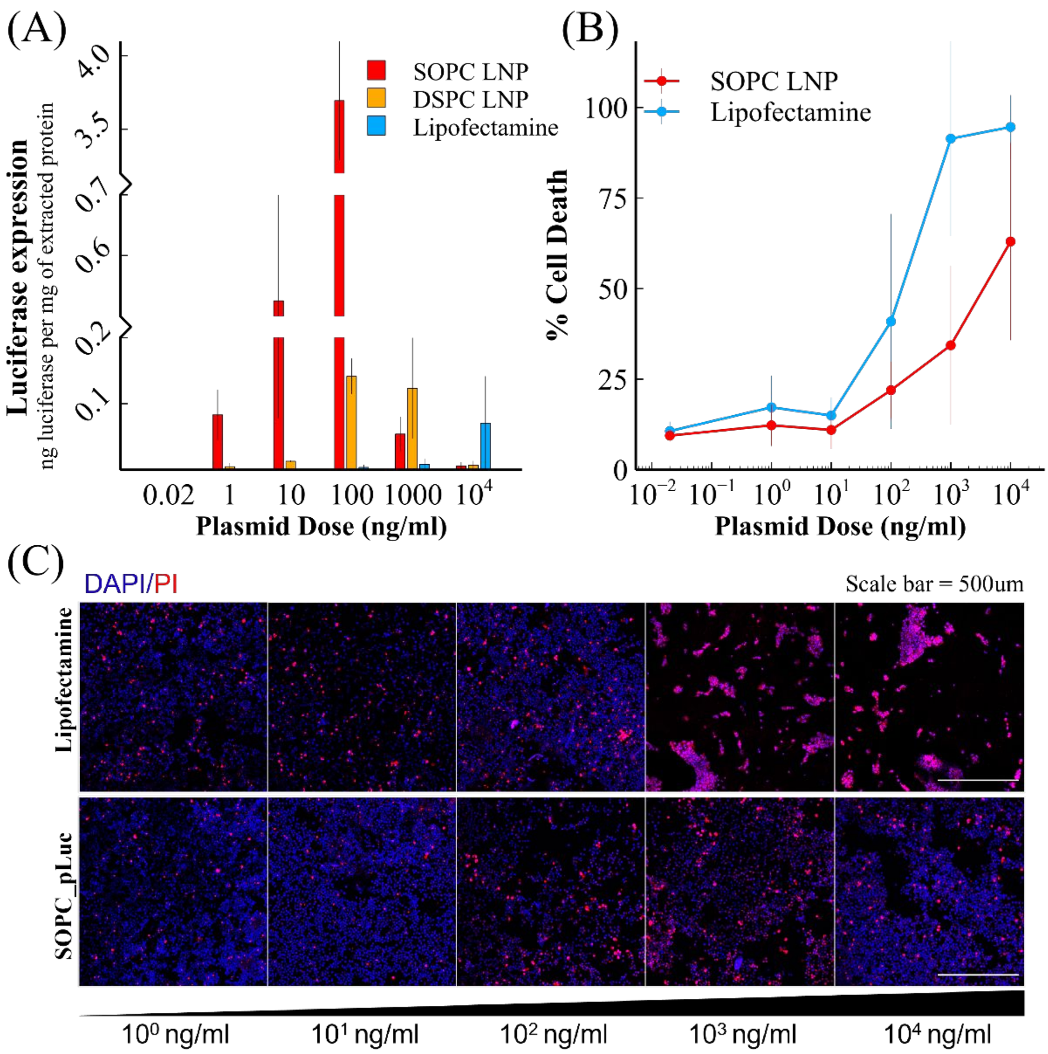

Figure S6. Concentration-dependent luciferase expression and cell cytotoxicity in Hela cells. Luciferase protein expression efficiency (A) and cytotoxicity (B) of SOPC_pLuc LNPs applied to Hela cells at the plasmid concentrations of 1, 20, 100, 1000 and 10000 ng/ml. Lipofectamine 2000 transfection reagent was used as a positive control. The cytotoxicity (% of dead cells) was calculated using ImageJ software from image analysis of confocal images (C). The Cell Viability Staining image is shown in (C). Confocal imaging of Hela cells showing all cells with nuclear DAPI stain (blue) and dead cells with Propidium iodide staining (red) for cells transfected with increasing concentrations of the plasmid.

SI 5. Fitting Models in SANS Data Analysis

SI 5.1 Core-shell model

The scattering intensity of the core-shell model was developed from the formulae of the dilute uniform sphere model. According to the small angle scattering (SAS) theory, the scattering intensity of a dilute uniform particle system can be described below

$$I(q) = NV^2(\Delta\rho)^2(F(Q))^2 + BKG \qquad S(3)$$

where *I* is the scattering intensity, which is a function of *q* (the scattering vector); BKG is the background; *N* is the number density of the particle, and *V* is the volume of a single particle. Thus, *NV* = *Φ*, which is the sample's volume fraction or concentration (volume/volume); Δρ is the neutron scattering length density difference between the particle and the solvent; F(q) is called the form factor. For a randomly oriented or centrosymmetric particle, *F(q)* is only related to the average radial

scattering length density distribution of the particle, *ρ(r)*. The *F(q)* can be expressed as in equation S(4) [2]

$$F(q) = \int 4\pi r^2 \rho(r) \frac{\sin(qr)}{qr} dr \quad S(4)$$

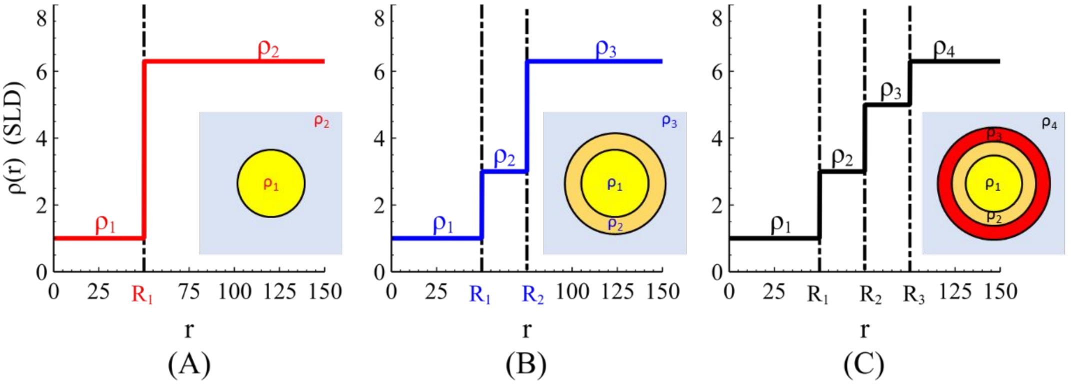

Figure S7. Exemplar radial SLD distributions and the corresponding schematics of (A) a uniform sphere particle, (B) a one shell sphere particle and (C) a sphere particle with two shells. The shells are concentric with the core here.

For a uniform sphere shown in **Figure S7** (A), *F(q) and I(q)* can be deduced from equation SS(4) as

$$F(q, R_1) = \frac{3(\sin(qR_1) - qR_1\cos(qR_1))}{(qR_1)^3} \quad S(5)$$

$$I(q) = NV^2(\rho_1 - \rho_2)^2 (F(q, R_1))^2 + BKG \quad S(6)$$

where ρ_1 and ρ_2 are the SLDs of the particle and the solvent as labelled in the schematic. *V* is the volume of the particle and can be calculated using the sphere volume function with the radius R_1. N is the number density of the particle.

For a sphere particle with a single shell as shown in **Figure S7** (B), the core-shell model can lead to

$$I(q) = N[(\rho_1 - \rho_2)V_1F(q, R_1) + (\rho_2 - \rho_3)V_2F(q, R_2)]^2 + BKG \quad S(7)$$

where ρ_1, ρ_2, ρ_3 are the SLDs of the core, shell and solvent as labelled in the schematic. V_1 and V_2 are the volumes of the spheres with radius of R_1 and R_2, respectively. In other words, V_1 is the volume of the core region, and V_2 is the total volume of the particle.

Similarly, the sphere particle with two shells shown in **Figure S7** (C) has the scattering intensity as

$$I(q) = N[(\rho_1 - \rho_2)V_1F(q, R_1) + (\rho_2 - \rho_3)V_2F(q, R_2) + (\rho_3 - \rho_4)V_3F(q, R_3)]^2 + BKG \quad S(8)$$

Furthermore, for a sphere particle with n shells, the function can be written as

$$I(q) = N\left[\sum_{1}^{n+1} (\rho_x - \rho_{x+1})V_xF(q, R_x)\right]^2 + BKG \quad S(9)$$

when x = n+1, R_{n+1} is the particle's radius, and V_{n+1} is the total volume of the particle.

SI 5.2 Teubner-Strey Model

The Teubner-Strey (T-S) model is a shape-independent model derived from Landau free energy theory to describe the scattering intensity from a binary component system with a quasi-periodic structure.[3]

The scattering intensity in the T-S model is expressed as

$$I_{Teubner-Strey}(q) = \frac{8\pi c_2\langle\sigma^2\rangle}{a + c_1q^2 + c_2q^4}\left(\frac{1}{2}\left(\frac{a}{c_2}\right)^{\frac{1}{2}} + \frac{1c_1}{4c_2}\right)^{\frac{1}{2}} \quad S(10)$$

where q is the scattering vector, $\langle\sigma^2\rangle$ is the contrast factor, which is

$$\langle\sigma^2\rangle = \varphi_{D2O} \cdot (1 - \varphi_{D2O}) \cdot (\rho_{D2O} - \rho_{Lipids})^2 \quad S(11)$$

where φ_{D2O} is the volume fraction of the water, ρ stands for scattering length density. In our work, the parameters (ρ_{Lipids}, ρ_{D2O} and φ_{D2O}) were used in both the core-shell and T-S models, consistent with data fitting.

The parameters a, c_1 and c_2 in Equation SS(10) are the coefficients of the Landau free energy. These three fitting parameters can be used to derive quantities describing the LNP system structure:[4]

$$d = 2\pi\left(\frac{1}{2}\left(\frac{a}{c_2}\right)^{\frac{1}{2}} - \left(\frac{c_1}{4c_2}\right)\right)^{\frac{-1}{2}} \quad S(12)$$

$$\varepsilon = \left(\frac{1}{2}\left(\frac{a}{c_2}\right)^{\frac{1}{2}} + \left(\frac{c_1}{4c_2}\right)\right)^{\frac{-1}{2}} \quad S(13)$$

$$\gamma = \frac{c_1}{\sqrt{4ac_2}} = \frac{\left(\frac{1}{\varepsilon}\right)^2 - \left(\frac{2\pi}{d}\right)^2}{\left(\frac{1}{\varepsilon}\right)^2 + \left(\frac{2\pi}{d}\right)^2} \quad S(14)$$

The 'periodicity length', d, is the averaged spatial period in the system covering the sum of the lengths of the two-component domains. The 'correlation length', ε, describes the distance over which the quasi-periodicity is lost. The 'amphiphile strength', γ, can be deduced from d and ε and is related to the type of structure formed by the system. Empirically, a disordered system has a $\gamma > 1$; a lamellar structure typically has $\gamma < -1$; $1 > \gamma > 0$ implies the formation of aggregates or micelles; $-1 < \gamma < 0$ corresponds to the adoption of non-lamellar structures.[4]

SI 5.3 SLDs and Binary Component System Simplification

The protonated lipids (including cholesterol) used to form the LNPs have the SLDs in the range from 0.09 to 0.3×10^{-6} Å^{-2}, as listed in **Table S3**. In the measurements under the contrast of protonated lipids and D_2O, the SLD differences between the lipids are far less than the SLD difference between the lipids and D_2O. Thus, the scattering signal is largely from the SLD contrast between the lipids and D_2O. Moreover, the nucleic acid-based plasmid has an SLD around 4.7×10^{-6} Å^{-2}, which is close to that of D_2O (6.35×10^{-6} Å^{-2}). Given that the plasmid content in the LNP is well below 3% (v/v) and DNA is strongly hydrated, we can neglect the plasmid's effect on the SLD of D_2O. Therefore, the LNPs can be simplified as a binary component system, containing all the lipids as the lipid component and the water (containing plasmid) as the solvent component.

Component		Molecular Volume ($Å^3$)	SLD (10^{-6} $Å^{-2}$)
MC3		1290	0.09
Cholesterol		630	0.21
DMG-PEG(2000)		3630	0.30
Protonated Helper Lipid	SOPC	1312	0.24
	POPC	1256	0.27
	DMPC	1100	0.28
	DPPC	1214	0.23
	DSPC	1327	0.18
Deuterated Helper Lipid	D62-DPPC	1214	5.54
	D31-POPC	1256	2.83
Solvent	D_2O	30	6.35
	H_2O	30	-0.56

Table S3. The SLDs (scattering length density) and molecular volumes of the lipids and solvents used in this work.

The T-S model was applied to the combined model based on this binary component system simplification as stated in SI 5.2. Also, benefitting from the binary component system simplification, the fraction of water and lipids in each region of the core-shell model can be deduced from the best-fit SLD via Equation SS(15).

$$SLD_{fitted} = \varphi_{D2O} \cdot SLD_{D2O} + (1 - \varphi_{D2O}) \cdot SLD_{lipids} \quad S(15)$$

where φ_{D2O} is the volume fraction of the water and φ_{lipids} = 1 - φ_{D2O}. The values of the averaged lipids' mixture SLD, SLD_{lipids}, are listed in **Table S4**. These values were calculated based on the assumption that the lipid mixture kept the same molar ratio in any given region or layer as the original value in the formulated sample (MC3: cholesterol: helper lipid: DMG-PEG (2000) were kept at 50:38.5:10:1.5, mol/mol). **Table S3** shows the SLDs of all the protonated lipids (including cholesterol) ranging from 0.09 to 0.3×10^{-6} $Å^{-2}$. Thus, the 'true' local SLD of the lipid mixture at any region of the LNP must be in the range of 0.09 to 0.3×10^{-6} $Å^{-2}$. The error between the 'true' local SLD of the lipid mixture and the averaged lipids' mixture SLD used in the calculation propagated to an error of ± 2% in the calculated water and lipid fractions of each LNP's region.

LNP Type	Molar proportion				Volume proportion				Average SLD of the Lipids Mixture
	MC3	Cholesterol	DMG-PEG(2000)	Helper Lipid	MC3	Cholesterol	DMG-PEG(2000)	Helper Lipid	
SOPC LNP	50.00%	38.50%	1.50%	10.00%	60.10%	22.60%	5.07%	12.23%	0.146
POPC LNP	50.00%	38.50%	1.50%	10.00%	60.42%	22.72%	5.10%	11.76%	0.149
DMPC LNP	50.00%	38.50%	1.50%	10.00%	61.31%	23.06%	5.18%	10.46%	0.148
DPPC LNP	50.00%	38.50%	1.50%	10.00%	60.65%	22.81%	5.12%	11.42%	0.144
DSPC LNP	50.00%	38.50%	1.50%	10.00%	60.02%	22.57%	5.07%	12.35%	0.139

Table S4. The lipid mixtures' averaged SLDs of the LNPs containing different helper lipids

SI 6. SANS Data Fitting and Analysis

SI 6.1 LNPs Containing Fully Protonated Lipids Measured in D_2O Buffer

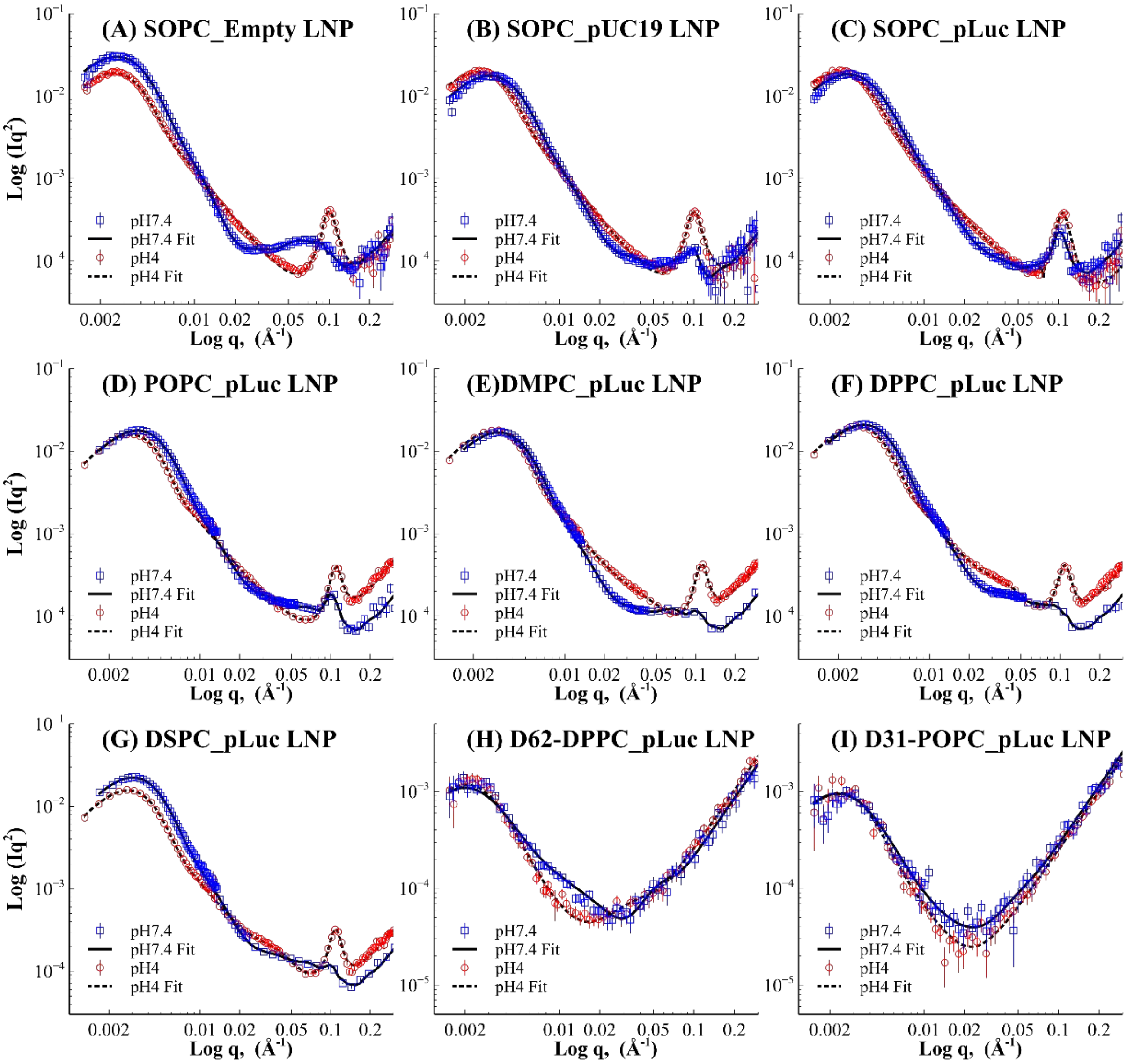

Figure S8. The SANS profiles of the LNPs containing protonated helper lipids with and without pLuc/pUC19 measured in D_2O buffer (A)(B)(C)(D)(E)(F)(G) and deuterated helper lipids measured in H_2O buffer (H)(I). The simulated profiles from the best-fit models are shown with the black lines.

Dimension							
pH	LNP Type	Core Radius (± 5Å)	Inner Shell Thickness (± 2Å)	Mid Shell Thickness (± 2Å)	Outer Shell Thickness (± 2Å)	LNP Total Radius* (± 6Å)	Schulz** Polydispersity
pH 7.4	SOPC_Empty	331	86	75	50	542	0.49
	SOPC_pUC19	295	80	30	50	455	0.48
	SOPC_pLuc	302	75	40	50	467	0.53
	POPC_pLuc	258	76	44	50	428	0.49
	DSPC_pLuc	301	73	45	50	468	0.46
	DPPC_pLuc	300	64	42	50	456	0.45
	DMPC_pLuc	275	85	41	50	451	0.49
pH 4.0	SOPC_Empty	494	-	-	50	544	0.36
	SOPC_pUC19	486	-	-	50	536	0.36
	SOPC_pLuc	480	-	-	50	530	0.41
	POPC_pLuc	440	-	-	50	490	0.33
	DSPC_pLuc	353	-	103	50	506	0.43
	DPPC_pLuc	359	-	97	50	506	0.42
	DMPC_pLuc	359	-	85	50	494	0.41

SLD					
pH	LNP Type	Core ($\pm 0.05\times10^{-6}$ Å^{-2})	Inner Shell ($\pm 0.05\times10^{-6}$ Å^{-2})	Mid Shell ($\pm 0.05\times10^{-6}$ Å^{-2})	Outer Shell ($\pm 0.05\times10^{-6}$ Å^{-2})
pH 7.4	SOPC_Empty	2.70	3.06	3.95	3.69
	SOPC_pUC19	1.35	1.93	3.37	1.88
	SOPC_pLuc	1.60	1.95	2.87	1.97
	POPC_pLuc	1.50	1.91	2.98	1.65
	DSPC_pLuc	1.70	2.08	3.11	1.92
	DPPC_pLuc	1.50	1.95	3.02	1.43
	DMPC_pLuc	1.40	1.82	3.21	1.70
pH 4.0	SOPC_Empty	3.50	-	-	3.37
	SOPC_pUC19	3.60	-	-	3.50
	SOPC_pLuc	3.50	-	-	3.04
	POPC_pLuc	3.50	-	-	3.05
	DSPC_pLuc	3.10	-	3.34	2.19
	DPPC_pLuc	3.20	-	3.40	2.17
	DMPC_pLuc	3.20	-	3.29	2.44

Table S5. The core-shells model parameters used to obtain the best-fits to the SANS data for the LNPs in the contrast of protonated lipids and D_2O. (Top) the dimension of each region including the radius of the core region and thicknesses of the shells. (Bottom) the SLD of each region.

*The total radius of an LNP was obtained by the sum of the dimension of each region.

**The Schulz distribution was used to describe the size distribution of the LNPs in each sample. The Schulz distribution is defined as[5,6]

$$f(r) = \left(\frac{Z+1}{R_{Mean}}\right)^{Z+1} \cdot r^{Z} \cdot exp\left[-\left(\frac{Z+1}{R_{Mean}}\right)r\right] \cdot \frac{1}{\Gamma(Z+1)} \qquad S(16)$$

where R_{Mean} is the mean of the sphere radius, Z is a parameter defining the width of the distribution; $\Gamma(x)$ is the Gamma function. The function approaches a delta function when $r = R_{Mean}$ as Z approaches infinity. The root mean square deviation from the mean is

$$\sigma = \frac{R_{Mean}}{(Z+1)^{1/2}} \qquad S(17)$$

Then, the Schulz polydispersity listed in Table S5 (Top) can be obtained as

$$PD_{Schulz} = \frac{\sigma}{R_{Mean}} = \frac{1}{(Z+1)^{1/2}} \qquad S(18)$$

The Schulz polydispersity is different from the PDI value measured by DLS. Schulz polydispersity describes the particle mean size and size distribution as shown in equation SS(18). In contrast, PDI obtained from DLS describes the distribution of autocorrelation function decay rates.[7]

T-S Model					
pH	LNP Type	Periodicity Length d (± 0.5Å)	Correlation Length ε (± 0.5Å)	Amphiphile Strength* γ (± 2%)	Normorlised T-S Scale Factor **
pH 7.4	SOPC_Empty	88.7	38.3	-0.761	0.263
	SOPC_pUC19	63.7	73.2	-0.962	0.199
	SOPC_pLuc	62.0	67.7	-0.958	0.413
	POPC_pLuc	63.2	66.8	-0.956	0.298
	DSPC_pLuc	65.0	51.7	-0.923	0.180
	DPPC_pLuc	66.7	48.6	-0.909	0.216
	DMPC_pLuc	64.0	47.1	-0.911	0.266
pH 4.0	SOPC_Empty	63.3	72.7	-0.962	0.481
	SOPC_pUC19	63.1	68.2	-0.957	0.465
	SOPC_pLuc	59.1	68.9	-0.963	0.502
	POPC_pLuc	58.0	72.7	-0.968	0.439
	DSPC_pLuc	58.5	77.5	-0.972	0.417
	DPPC_pLuc	58.7	71.3	-0.966	0.450
	DMPC_pLuc	56.9	73.4	-0.970	0.489

Table S6. The parameters obtained in the T-S model. Values of periodicity length d and correlation length ε were obtained from the best-fit results.

*The values of amphiphile strength γ were calculated from d and ε using Equation SS(14).

**The scale factor of the T-S model was normalized by the lipid concentration of the sample.

Volume						
pH	LNP Type	Core (± 0.03×10^{8}Å^3)	Inner Shell (± 0.04×10^{8}Å^3)	Mid Shell (± 0.04×10^{8}Å^3)	Outer Shell (± 0.06×10^{8}Å^3)	LNP Total Volume* (± 0.09×10^{8}Å^3)
pH 7.4	SOPC_Empty	1.52	1.52	1.94	1.68	6.67
	SOPC_pUC19	1.08	1.13	0.57	1.16	3.94
	SOPC_pLuc	1.15	1.09	0.79	1.23	4.26
	POPC_pLuc	0.72	0.85	0.70	1.02	3.29
	DSPC_pLuc	1.14	1.04	0.89	1.24	4.30
	DPPC_pLuc	1.13	0.89	0.79	1.17	3.98
	DMPC_pLuc	0.87	1.08	0.75	1.14	3.84
pH 4.0	SOPC_Empty	5.04	-	-	1.69	6.73
	SOPC_pUC19	4.80	-	-	1.64	6.44
	SOPC_pLuc	4.63	-	-	1.60	6.24
	POPC_pLuc	3.57	-	-	1.36	4.93
	DSPC_pLuc	1.84	-	2.14	1.46	5.43
	DPPC_pLuc	1.94	-	2.02	1.45	5.41
	DMPC_pLuc	1.94	-	1.73	1.38	5.05

Table S7. The volumes of different regions were calculated from the fitted dimensions of the SANS results.

*The LNP total volume is the sum of the volumes of individual regions. The volume of each region can be calculated from the fitted dimension parameters listed in **Table S5** (Top).

The volume of the core region was calculated using the sphere volume function below

$$V_{core} = \frac{4}{3}\pi r_c^3 \qquad S(19)$$

where V_{core} is the volume of the core and r_c is its radius. The volume of each shell was calculated as

$$V_{shell} = \frac{4}{3}\pi\left(r_e^3 - r_i^3\right) \qquad S(20)$$

where V_{shell} is the volume of a shell with an external radius r_e and internal radius r_i.

Water Fraction Percentage					
pH	LNP Type	Core	Inner Shell	Mid Shell	Outer Shell
pH 7.4	SOPC_Empty	41.9%	47.7%	62.4%	58.1%
	SOPC_pUC19	19.8%	29.3%	52.8%	28.4%
	SOPC_pLuc	23.9%	29.6%	44.6%	29.9%
	POPC_pLuc	22.3%	29.0%	46.5%	24.6%
	DSPC_pLuc	25.5%	31.8%	48.6%	29.1%
	DPPC_pLuc	22.3%	29.7%	47.1%	21.0%
	DMPC_pLuc	20.6%	27.5%	50.2%	25.5%
pH 4.0	SOPC_Empty	55.0%	-	-	52.9%
	SOPC_pUC19	56.6%	-	-	55.0%
	SOPC_pLuc	55.0%	-	-	47.4%
	POPC_pLuc	55.0%	-	-	47.6%
	DSPC_pLuc	48.4%	-	52.3%	33.5%
	DPPC_pLuc	50.1%	-	53.4%	33.2%
	DMPC_pLuc	50.1%	-	51.6%	37.6%

Lipid Fraction Percentage					
pH	LNP Type	Core	Inner Shell	Mid Shell	Outer Shell
pH 7.4	SOPC_Empty	58.1%	52.3%	37.6%	41.9%
	SOPC_pUC19	80.2%	70.7%	47.2%	71.6%
	SOPC_pLuc	76.1%	70.4%	55.4%	70.1%
	POPC_pLuc	77.7%	71.0%	53.5%	75.4%
	DSPC_pLuc	74.5%	68.2%	51.4%	70.9%
	DPPC_pLuc	77.7%	70.3%	52.9%	79.0%
	DMPC_pLuc	79.4%	72.5%	49.8%	74.5%
pH 4.0	SOPC_Empty	45.0%	-	-	47.1%
	SOPC_pUC19	43.4%	-	-	45.0%
	SOPC_pLuc	45.0%	-	-	52.6%
	POPC_pLuc	45.0%	-	-	52.4%
	DSPC_pLuc	51.6%	-	47.7%	66.5%
	DPPC_pLuc	49.9%	-	46.6%	66.8%
	DMPC_pLuc	49.9%	-	48.4%	62.4%

Table S8. Percentages of water and lipid fractions in each region of LNP.

The percentages of water and lipid fractions were calculated from the fitted SLDs listed in **Table S5** (bottom). The calculation is based on equation SS(15).

Lipid Volume						
**pH**	**LNP Type**	**Core ($\pm 0.03\times10^{8}$Å^{3})**	**Inner Shell ($\pm 0.04\times10^{8}$Å^{3})**	**Mid Shell ($\pm 0.04\times10^{8}$Å^{3})**	**Outer Shell ($\pm 0.06\times10^{8}$Å^{3})**	**Total* ($\pm 0.09\times10^{8}$Å^{3})**
pH 7.4	SOPC_Empty	0.88	0.80	0.73	0.70	3.11
	SOPC_pUC19	0.86	0.80	0.27	0.83	2.76
	SOPC_pLuc	0.87	0.77	0.44	0.86	2.94
	POPC_pLuc	0.56	0.60	0.37	0.77	2.31
	DSPC_pLuc	0.85	0.71	0.46	0.88	2.89
	DPPC_pLuc	0.88	0.63	0.42	0.92	2.84
	DMPC_pLuc	0.69	0.78	0.37	0.85	2.70
pH 4.0	SOPC_Empty	2.27	-	-	0.80	3.06
	SOPC_pUC19	2.08	-	-	0.74	2.82
	SOPC_pLuc	2.08	-	-	0.84	2.93
	POPC_pLuc	1.61	-	-	0.71	2.32
	DSPC_pLuc	0.95	-	1.02	0.97	2.94
	DPPC_pLuc	0.97	-	0.94	0.97	2.88
	DMPC_pLuc	0.97	-	0.84	0.86	2.66

Water Volume						
**pH**	**LNP Type**	**Core ($\pm 0.03\times10^{8}$Å^{3})**	**Inner Shell ($\pm 0.04\times10^{8}$Å^{3})**	**Mid Shell ($\pm 0.04\times10^{8}$Å^{3})**	**Outer Shell ($\pm 0.06\times10^{8}$Å^{3})**	**Total* ($\pm 0.09\times10^{8}$Å^{3})**
pH 7.4	SOPC_Empty	0.64	0.73	1.21	0.98	3.55
	SOPC_pUC19	0.21	0.33	0.30	0.33	1.18
	SOPC_pLuc	0.27	0.32	0.35	0.37	1.32
	POPC_pLuc	0.16	0.25	0.33	0.25	0.98
	DSPC_pLuc	0.29	0.33	0.43	0.36	1.41
	DPPC_pLuc	0.25	0.26	0.37	0.25	1.13
	DMPC_pLuc	0.18	0.30	0.37	0.29	1.14
pH 4.0	SOPC_Empty	2.77	-	-	0.90	3.67
	SOPC_pUC19	2.72	-	-	0.90	3.62
	SOPC_pLuc	2.55	-	-	0.76	3.31
	POPC_pLuc	1.96	-	-	0.65	2.61
	DSPC_pLuc	0.89	-	1.12	0.49	2.50
	DPPC_pLuc	0.97	-	1.08	0.48	2.53
	DMPC_pLuc	0.97	-	0.89	0.52	2.38

Table S9. Volumes of water fraction and lipid fraction in each region of LNP.

The volumes of water and lipid in each region of LNP can be calculated as the product of each region volume (listed in Table S9) and the corresponding volume fraction (listed in Table S8).

*The total lipid volume of each LNP was calculated as the sum of the lipid volume in each region. The total water volume was calculated similarly.

An averaged water volume fraction ($\bar{f}$) can be calculated for each LNP using equation SS(21) as shown below

$$\bar{f} = \frac{\sum_{i=1}^{n} (V_i \times f_i)}{\sum_{i=1}^{n} V_i} \qquad S(21)$$

where n indicates the number of regions in the LNP and i is the region indicator. For example, in the case of SOPC_pLuc LNP at pH 7.4, regions 1, 2, 3, 4 correspond to the regions of Core, Shell 1 (Inner Shell), Shell 2 (Mid Shell) and Shell 3 (Outer Shell), respectively. V_i is the volume of region i while f_i is the water volume fraction of region i. The averaged lipid volume fraction in each region is simply $1 - \bar{f}$. The results are listed in **Table S10**.

Averaged Water Volume Fraction		
LNP Type	pH 7.4	pH 4
SOPC_Empty	53.3%	54.5%
SOPC_pUC19	29.9%	56.2%
SOPC_pLuc	30.9%	53.1%
POPC_pLuc	29.9%	53.0%
DSPC_pLuc	32.8%	46.0%
DPPC_pLuc	28.5%	46.8%
DMPC_pLuc	29.8%	47.2%

Averaged Lipid Volume Fraction		
LNP Type	pH 7.4	pH 4
SOPC_Empty	46.7%	45.5%
SOPC_pUC19	70.1%	43.8%
SOPC_pLuc	69.1%	46.9%
POPC_pLuc	70.1%	47.0%
DSPC_pLuc	67.2%	54.0%
DPPC_pLuc	71.5%	53.2%
DMPC_pLuc	70.2%	52.8%

Table S10. The averaged water and lipid volume fractions in each region of the LNP at pH 7.4 and pH 4.

SI 6.2 LNPs Containing Deuterated Helper Lipids

LNPs containing deuterated helper lipids (D62-DPPC and D31-POPC) were manufactured using the method described previously. The SANS measurements of the LNPs containing deuterated helper lipids were undertaken in H_2O buffer at pH 7.4 and pH 4, respectively. The measured SANS profiles are shown in **Figure S8** (H) for D62-DPPC and **Figure S8** (I) for D31-POPC. A simultaneous fitting analysis was carried out to the two SLD-contrasts obtained: H-LNP (LNP containing protonated lipids) measured in D_2O buffer, D-LNP (LNP containing protonated MC3, cholesterol and PEG lipid but deuterated helper lipid) measured in H_2O buffer. The best-fit models are shown as the black lines in **Figure S8**, with the parameters obtained listed in **Table S11**. In the simultaneous fitting analysis, the core radii and shell thicknesses are consistent for each LNP at each pH under the two SLD-contrasts investigated. The parameters for the contrast of 'H-LNP in D_2O' are also listed in **Table S5** for easy comparison.

LNP	pH	Contrast	Core Radius (± 5Å)	Inner Shell Thickness (± 2Å)	Mid Shell Thickness (± 2Å)	Outer Shell Thickness (± 2Å)	Core SLD (± 0.05×10^{-6} Å^{-2})	Inner Shell SLD (± 0.05×10^{-6} Å^{-2})	Mid Shell SLD (± 0.05×10^{-6} Å-2)	Outer Shell SLD (± 0.05×10^{-6} Å^{-2})
POPC_pLuc	7.4	H-LNP in D2O	258	76	44	50	1.50	1.91	2.98	1.65
		D-LNP in H2O	258	76	44	50	0.19	0.26	0.02	0.80
	4	H-LNP in D2O	440	-	-	50	3.50	-	-	3.05
		D-LNP in H2O	440	-	-	50	0.06	-	-	0.05
DPPC_pLuc	7.4	H-LNP in D2O	300	64	42	50	1.50	1.95	3.02	1.43
		D-LNP in H2O	300	64	42	50	-0.02	0.33	-0.18	1.12
	4	H-LNP in D2O	359	-	97	50	3.20	-	3.40	2.17
		D-LNP in H2O	359	-	97	50	0.02	-	-0.17	0.69

Table S11. The best-fit parameters were obtained from the simultaneous fitting analysis to the POPC_pLuc LNP and DPPC_pLuc LNP at pH 4 and 7.4.

Benefitting from the combined uses of hydrogenated and partially deuterated helper lipids (D62-DPPC and D31-POPC), the helper lipids can be distinguished from the other lipid components. Thus, the LNP can be regarded as a 3-component system under such combined deuterium labelling schemes, containing water, deuterated helper lipid and the protonated lipid mixture (protonated MC3, cholesterol and DMG-PEG(2000)). The SLD of each region under the 'H-LNP in D_2O buffer' contrast can be expressed as

$$SLD_{H-LNP} = \varphi_w \cdot SLD_{D2O} + \varphi_{Helper} \cdot SLD_{H-Helper} + (1 - \varphi_w - \varphi_{Helper}) \cdot SLD_{Mixtures} \quad S(22)$$

The SLD of each region under the 'D-LNP in H_2O buffer' contrast can be expressed as

$$SLD_{D-LNP} = \varphi_w \cdot SLD_{H2O} + \varphi_{Helper} \cdot SLD_{D-Helper} + (1 - \varphi_w - \varphi_{Helper}) \cdot SLD_{Mixtures} \quad S(23)$$

where SLD_{H-LNP} and SLD_{D-LNP} are the SLDs of each region under the two SLD-contrasts involving hydrogenated and deuterated lipids, obtained from the simultaneous fitting analysis; SLD_{D2O}, SLD_{H2O} are the SLDs of D_2O and H_2O, listed in **Table S3**; $SLD_{H-Helper}$ and $SLD_{D-Helper}$ are the SLDs of the protonated and deuterated helper lipids. The SLDs of D62-DPPC, D31-POPC, protonated POPC and DPPC were given in **Table S3**; $SLD_{Mixtures}$ is the SLD of the lipid mixture containing protonated MC3, cholesterol and DMG-PEG(2000). $SLD_{Mixtures}$ is 0.13×10^{-6} Å^{-2} in this study, calculated from **Table S4**; φ_w and φ_{Helper} are the volume fractions of water and helper lipid.

Since φ_w and φ_{Helper} are the only two unknown variables, the unique solutions for these two variables can be obtained by combining equations SS(22) and SS(23). The obtained volume fractions of water and helper lipid are given in **Table S12**.

pH	LNP Type	Water Volume Fraction				Helper Lipid Volume Fraction			
		Core	Inner Shell	Mid Shell	Outer Shell	Core	Inner Shell	Mid Shell	Outer Shell
7.4	POPC_pLuc	22.3%	29.0%	46.5%	24.6%	3.9%	6.0%	4.0%	15.5%
	DPPC_pLuc	22.3%	29.7%	47.1%	21.0%	0.1%	7.4%	0.3%	21.0%
4	POPC_pLuc	55.0%	-	-	47.6%	5.6%	-	-	4.6%
	DPPC_pLuc	50.1%	-	53.4%	33.2%	4.4%	-	1.3%	14.6%

Table S12. The combined analysis of the SANS profiles measured under the two contrasts led to water volume fraction and helper lipid volume fraction for each region in the POPC and DPPC LNPs at pH 7.4 and pH 4.

SI 7. SD_{water}, Standard Deviation (SD) of Water Volume Fraction in LNP

The standard deviation (SD) of the water fraction in an LNP is calculated from equation SS(24). This equation is adapted from the standard deviation function for frequency distribution, meaning that the deviation in water fraction is weighted by the volume proportion of each region.

$$SD_{water} = \sqrt{\frac{\sum_{i=1}^{n} \left(V_i \times (f_i - \bar{f})^2\right)}{\sum_{i=1}^{n} V_i}} \qquad S(24)$$

where n shows the number of regions in the LNP and i is the region indicator. For example, in the case of SOPC_pLuc LNP at pH 7.4, regions 1, 2, 3, 4 correspond to the regions of Core, Shell 1 (Inner Shell), Shell 2 (Mid Shell) and Shell 3 (Outer Shell), respectively. V_i is the volume of region i, while f_i is its corresponding water volume fraction. $\bar{f}$ is the average water fraction of the LNP, which is calculated using equation S(21) and listed in **Table S10** for each LNP. $(\sum_{i=1}^{n} V_i)$ is the total volume of the LNP. The calculation of the lipid volume and water volume fraction in each region, the averaged water volume fraction and total LNP volume in an LNP are described in SI 6. For example, the variables used in the calculation of SD_{water} for the SOPC_pLuc LNP at pH 7.4 are shown in **Table S13**. The SD_{water} results of all the LNPs at pH 7.4 and pH 4 are listed in **Table S14**.

Variable	Label	Values			
Number of Regions	n	4			
Region Indx	i	1	2	3	4
Region	-	Core	Inner Shell	Mid Shell	Outer Shell
Volume (10^8Å^3)	V_i	1.15	1.09	0.79	1.23
Total Volume (10^8Å^3)	$\sum_{i=1}^{n} V_i$	4.26			
Water Volume Fraction	f_i	23.9%	29.6%	44.6%	29.9%
Averaged Water Volume Fraction	$\bar{f}$	30.9%			
Sandard deviation	SD_{water}	7.0%			

Table S13. The variables used in the calculation of SD_{water} for the SOPC_pLuc LNP at pH 7.4.

SD_{water}		
LNP Type	pH 7.4	pH 4
SOPC_Empty	8.2%	0.9%
SOPC_pUC19	10.3%	0.7%
SOPC_pLuc	7.0%	3.3%
POPC_pLuc	9.0%	3.3%
DSPC_pLuc	8.3%	7.7%
DPPC_pLuc	9.8%	8.4%
DMPC_pLuc	10.3%	5.9%

Table S14. The SD_{water} values were calculated for all the LNPs investigated at pH 7.4 and 4.

In this work, SD_{water} evaluates the variation associated with the water volume fraction estimated in each region of the LNP. It measures how much the water volume fraction of each region is spread out around the averaged water volume fraction. The standard deviation (SD) of the LNP's lipid fraction, SD_{lipid} can be calculated following the same method. Because lipid and water volume fractions are always complementary in a binary component system, SD_{water} and SD_{lipid} are always equal for each LNP.

SI 8. LNP's Structural Feature Space (LSF) and Similarity Analysis

SI 8.1 LNP's Structural Feature Space

Since multiple variables were required to describe an LNP at a certain pH, an LNP's structural feature space (LSF) is used to simplify the analysis of the experimental results. LSF contain 15 coordinates, and each represents an essential structural feature of the LNP. The 15 structural features include the volume and water volume fraction of each region, the total volume and averaged water fraction of the entire LNP, SD_{water} showing the inhomogeneity of water distribution and the 4 T-S model parameters describing the small-length-scale nanostructures. Thus, each LNP has a unique position in the vector space of LSF. The relative position of given coordinates in LSF for a pair of LNPs can be used to analyze their similarity as discussed later.

An original parameter space (OPS) is first obtained by combining the 15 structural feature parameters (p1~p15) in a certain order as the coordinates of OPS, as shown in **Table S15**. The position or value in each coordinate can be assigned from the experimental data, e.g., see the assignments of the p1~p15 values of SOPC_pLuc LNP at pH 7.4 in OPS in the top row in **Table S15**. Subsequently, the LSF space can be obtained by normalizing OPS coordinates with the respective SOPC_pLuc LNP values at pH 7.4. Thus, the position of SOPC_pLuc LNP at pH 7.4 in LSF is obtained by the normalization as the **1**-vector, (1, 1, 1, 1, 1, 1, 1, 1, 1, 1, 1, 1, 1, 1, 1), as shown in the top row in **Table S16**. The same approach applies to data of the other LNPs at both pH 7.4 and 4. The normalization process makes all the coordinates of LSF unitless and equal-weighted in the similarity analysis. The positions of LNPs in LSF are given in **Table S16**.

		Volume ($\times 10^8 Å^3$)					Water Volume Fraction						T-S Parameter			
Parameters		p1	p2	p3	p4	p5	p6	p7	p8	p9	p10	p11	p12	p13	p14	p15
pH	LNP Type	Core	Inner Shell	Mid Shell	Outer Shell	Total Volume	Core	Inner Shell	Mid Shell	Outer Shell	Average	SD_{water}	Periodicity Length	Correlation Length	Amphiphile Strength	Normorlised T-S Scale Factor
7.4	SOPC_p-Med-Vac-3	1.15	1.09	0.79	1.23	4.26	23.9%	29.6%	44.6%	29.9%	30.9%	7.0%	62.0	67.7	-0.958	0.413
	POPC_p-Med-Vac-3	0.72	0.85	0.70	1.02	3.29	22.3%	29.0%	46.5%	24.6%	29.9%	9.0%	63.2	66.8	-0.956	0.298
	DSPC_p-Med-Vac-3	1.14	1.04	0.89	1.24	4.30	25.5%	31.8%	48.6%	29.1%	32.8%	8.3%	65.0	51.7	-0.923	0.180
	DPPC_p-Med-Vac-3	1.13	0.89	0.79	1.17	3.98	22.3%	29.7%	47.1%	21.0%	28.5%	9.8%	66.7	48.6	-0.909	0.216
	DMPC_p-Med-Vac-3	0.87	1.08	0.75	1.14	3.84	20.6%	27.5%	50.2%	25.5%	29.8%	10.3%	64.0	47.1	-0.911	0.266
4	SOPC_p-Med-Vac-3	4.63	0	0	1.60	6.24	55.0%	0.0%	0.0%	47.4%	53.1%	3.3%	59.1	68.9	-0.963	0.502
	POPC_p-Med-Vac-3	3.57	0	0	1.36	4.93	55.0%	0.0%	0.0%	47.6%	53.0%	3.3%	58.0	72.7	-0.968	0.439
	DSPC_p-Med-Vac-3	1.84	2.14	0	1.46	5.43	48.4%	52.3%	0.0%	33.5%	46.0%	7.7%	58.5	77.5	-0.972	0.417
	DPPC_p-Med-Vac-3	1.94	2.02	0	1.45	5.41	50.1%	53.4%	0.0%	33.2%	46.8%	8.4%	58.7	71.3	-0.966	0.450
	DMPC_p-Med-Vac-3	1.94	1.73	0	1.38	5.05	50.1%	51.6%	0.0%	37.6%	47.2%	5.9%	56.9	73.4	-0.970	0.489

Table S15. The positions of LNPs with different helper lipids at pH 7.4 and pH 4, in the original parameters space (OPS).

Dimensions		p'1	p'2	p'3	p'4	p'5	p'6	p'7	p'8	p'9	p'10	p'11	p'12	p'13	p'14	p'15
7.4	SOPC_pLuc	1.000	1.000	1.000	1.000	1.000	1.000	1.000	1.000	1.000	1.000	1.000	1.000	1.000	1.000	1.000
	POPC_pLuc	0.627	0.778	0.883	0.834	0.773	0.932	0.978	1.043	0.825	0.966	1.283	1.020	0.986	0.997	0.721
	DSPC_pLuc	0.991	0.959	1.120	1.008	1.011	1.068	1.072	1.089	0.973	1.061	1.193	1.049	0.763	0.963	0.435
	DPPC_pLuc	0.981	0.819	0.998	0.954	0.935	0.932	1.002	1.057	0.704	0.921	1.406	1.077	0.717	0.948	0.523
	DMPC_pLuc	0.761	0.992	0.943	0.931	0.903	0.863	0.930	1.125	0.853	0.962	1.478	1.033	0.695	0.950	0.645
4	SOPC_pLuc	4.032	0.000	0.000	1.307	1.466	2.301	0.000	0.000	1.588	1.715	0.472	0.955	1.017	1.005	1.217
	POPC_pLuc	3.106	0.000	0.000	1.109	1.158	2.301	0.000	0.000	1.594	1.712	0.471	0.936	1.074	1.010	1.064
	DSPC_pLuc	1.601	1.963	0.000	1.187	1.277	2.027	1.766	0.000	1.122	1.486	1.103	0.944	1.144	1.014	1.010
	DPPC_pLuc	1.686	1.860	0.000	1.184	1.272	2.096	1.804	0.000	1.112	1.513	1.194	0.947	1.052	1.008	1.089
	DMPC_pLuc	1.684	1.587	0.000	1.127	1.186	2.096	1.742	0.000	1.260	1.526	0.845	0.919	1.083	1.012	1.184

Table S16. The positions of LNPs with different helper lipids at pH 7.4 and pH 4, in the LNP's structural feature space (LSF).

SI 8.2 Similarity Analysis

The similarity between two LNPs can be evaluated by calculating the Euclidean distance between the LNP pair in LSF by following equation SS(25):

$$DSF = \sqrt{\sum_{i=1}^{15} (LNP\_1_i - LNP\_2_i)^2} \quad S(25)$$

where DSF is the Euclidean distance between LNP_1 and LNP_2 in LSF. LNP_1_i is the ith element of LNP_1's position in LSF. DSF is a non-negative real number, and it is equal to 0 when an LNP is compared with itself. The larger DSF indicates less similarity between the two LNPs under comparison. For example, at pH 4, DSF between SOPC_pLuc and POPC_pLuc LNPs is 0.998, while it is 3.700 between SOPC_pLuc and DSPC_pLuc LNPs. Therefore, the POPC_pLuc LNP is similar to SOPC_pLuc LNP, but quite different to the DSPC_pLuc LNP.

SI 9. Pearson Correlation Coefficient, PCC

The Pearson correlation coefficient (PCC) is a well-established indicator to measure the strength of linear correlation between two data sets. PCC always has a value between -1 to 1, while the two boundaries of -1 and 1 indicate that the two data sets have a perfect negative and positive linear relationship. There is no correlation between the data sets when PCC is equal to 0. PCC can be calculated from the following equation

$$PCC_{X,Y} = \frac{cov(X,Y)}{\sigma_X \cdot \sigma_Y} \quad S(26)$$

where *X*, *Y* stand for the data sets *X* and *Y*, and σ_X and σ_Y are their standard deviations, respectively. Function $cov(X,Y)$ describes the covariance of *X*, *Y* and is expressed as

$$cov(X,Y) = E[(X - \bar{x}) \cdot (Y - \bar{y})] \quad S(27)$$

where *E* stands for expectation and $\bar{x}$ and $\bar{y}$ are the means of *X* and *Y*, respectively.

References:

(1) Santos, J. L.; Ren, Y.; Vandermark, J.; Archang, M. M.; Williford, J.-M.; Liu, H.-W.; Lee, J.; Wang, T.-H.; Mao, H.-Q. Continuous Production of Discrete Plasmid DNA-Polycation Nanoparticles Using Flash Nanocomplexation. *Small* **2016**, *12* (45), 6214–6222. https://doi.org/10.1002/smll.201601425.

(2) A. Guinier; G. Fournet. *Small Angle Scattering of X-Rays*; Wiley: New York, USA, 1955.

(3) Teubner, M.; Strey, R. Origin of the Scattering Peak in Microemulsions. *J. Chem. Phys.* **1987**, *87* (5), 3195–3200. https://doi.org/10.1063/1.453006.

(4) Cabry, C. P.; D'Andrea, L.; Shimizu, K.; Grillo, I.; Li, P.; Rogers, S.; Bruce, D. W.; Canongia Lopes, J. N.; Slattery, J. M. Exploring the Bulk-Phase Structure of Ionic Liquid Mixtures Using Small-Angle Neutron Scattering. *Faraday Discuss.* **2018**, *206* (0), 265–289. https://doi.org/10.1039/C7FD00167C.

(5) Kotlarchyk, M.; Chen, S. Analysis of Small Angle Neutron Scattering Spectra from Polydisperse Interacting Colloids. *J. Chem. Phys.* **1983**, *79* (5), 2461–2469. https://doi.org/10.1063/1.446055.

(6) Kotlarchyk, M.; Stephens, R. B.; Huang, J. S. Study of Schultz Distribution to Model Polydispersity of Microemulsion Droplets. *J. Phys. Chem.* **1988**, *92* (6), 1533–1538. https://doi.org/10.1021/j100317a032.

(7) King, S.; Washington, C.; Heenan, R. Polyoxyalkylene Block Copolymers Adsorbed in Hydrocarbon and Fluorocarbon Oil-in-Water Emulsions. *Phys. Chem. Chem. Phys.* **2005**, *7* (1), 143. https://doi.org/10.1039/b414175j.
